# Supplementary material for: Diurnal cortisol and mental well-being in middle and older age: evidence from four cohort studies
Source: BMJ Open. 2017 Oct 12;7(10):e016085. doi: 10.1136/bmjopen-2017-016085 (PMC5652457; doi:10.1136/bmjopen-2017-016085)
Supplement: Author's manuscript [file bmjopen-2017-016085.draft_revisions.pdf]

BMJ Open

BMJ Open is committed to open peer review. As part of this commitment we make the peer review history of every article we publish publicly available.

When an article is published we post the peer reviewers' comments and the authors' responses online. We also post the versions of the paper that were used during peer review. These are the versions that the peer review comments apply to.

The versions of the paper that follow are the versions that were submitted during the peer review process. They are not the versions of record or the final published versions. They should not be cited or distributed as the published version of this manuscript.

BMJ Open is an open access journal and the full, final, typeset and author-corrected version of record of the manuscript is available on our site with no access controls, subscription charges or pay-per-view fees (http://bmjopen.bmj.com).

If you have any questions on BMJ Open's open peer review process please email info.bmjopen@bmj.com

Diurnal cortisol and mental wellbeing in middle and older age: evidence from four cohort studies

Journal:	*BMJ Open*
Manuscript ID	bmjopen-2017-016085
Article Type:	Research
Date Submitted by the Author:	30-Jan-2017

Complete List of Authors:	Stafford, Mai; University College London, MRC Unit for Lifelong Health and Ageing<br>Ben-Shlomo, Yoav; University of Bristol,<br>Cooper, Cyrus; University of Southampton and Southampton University Hospitals NHS Trust, MRC Lifecourse Epidemiology Unit<br>Gale, Catharine; MRC Epidemiology Resource Centre; University of Edinburgh, Department of Psychology<br>Gardner, Mike; University of Oxford, Nuffield Department of Population Health<br>Geoffroy, Marie-Claude; McGill University, McGill Group for Suicide Studies at Douglas Mental Health University Institute; McGill University, Department of Psychiatry<br>Power, Christine<br>Kuh, Diana; MRC Unit for Lifelong Health and Ageing at UCL,<br>Cooper, Rachel; UCL, MRC Unit for Lifelong Health and Ageing at UCL

**Primary Subject Heading**:	Epidemiology
Secondary Subject Heading:	Mental health
Keywords:	meta-analysis, individual participant data, positive psychology

Diurnal cortisol and mental wellbeing in middle and older age: evidence from four cohort studies

Authors information

Mai Stafford[1], Yoav Ben-Shlomo[2], Cyrus Cooper[3], Catharine Gale[3,4], Michael P. Gardner[5], Marie-Claude Geoffroy[6], Chris Power[7], Diana Kuh[1], Rachel Cooper[1]

[1]MRC Unit for Lifelong Health and Ageing at UCL, UCL, London, UK

[2]School of Social and Community Medicine, University of Bristol, Bristol, UK

[3]MRC Lifecourse Epidemiology Unit, University of Southampton, Southampton, UK

[4]Department of Psychology, University of Edinburgh, Edinburgh, UK

[5]Nuffield Department of Population Health, University of Oxford, Oxford, UK

[6]McGill Group for Suicide Studies at Douglas Mental Health University Institute; and Department of Psychiatry, McGill University, Montréal, Canada

[7]Population, Policy and Practice Programme, UCL Great Ormond Street Institute of Child Health, UCL, London, UK

Correspondence to: Dr Mai Stafford, MRC Unit for Lifelong Health and Ageing at UCL, 33 Bedford Place, London, WC1B 5JU; email: m.stafford@ucl.ac.uk; tel: +44 207 570 5712

Word count: 3040

Number of figures and tables: 3 figures, 2 tables

For peer review only

Diurnal cortisol and mental wellbeing in middle and older age: evidence from four cohort studies

ABSTRACT

Objectives: We conducted individual participant meta-analysis to test the hypothesis that cortisol patterns indicative of dysregulated HPA-axis functioning would be prospectively associated with poorer wellbeing at follow-up.

Setting: Four large UK-based cohort studies

Participants: Those providing valid salivary or serum cortisol samples (n=7515 for morning cortisol; n=1756 for evening cortisol) at baseline (age 44-82) and wellbeing data on the Warwick Edinburgh Mental Wellbeing Scale at follow-up (0-8 years) were included.

Results: Wellbeing was not associated with morning cortisol or diurnal slope though a borderline association with evening cortisol was found. Adjusting for sex and follow-up time, each 1 standard deviation increase in evening cortisol was associated with a -0.47 (95% CI -1.00, 0.05) point lower WEMWBS. This was attenuated by adjustment for body mass index, smoking and socioeconomic position. Between-study heterogeneity was low.

Conclusions: This study does not support the hypothesis that diurnal cortisol is prospectively associated with wellbeing up to 8 years later. However, replication in prospective studies with cortisol samples over multiple days is required.

Keywords: meta-analysis; individual participant data; positive psychology;

For peer review only

For peer review only - http://bmjopen.bmj.com/site/about/guidelines.xhtml

Strengths

- Individual participant meta-analysis based on 1612-7515 participants with cortisol data
- Prospective study with up to 8 years of follow-up
- Validated mental wellbeing instrument based on 14 items capturing hedonic and eudaimonic components harmonized across cohorts

Limitations

- Salivary cortisol samples taken over 1 or 2 days; up to a maximum of 4 times per day
- Actual waking time not recorded in all studies

For peer review only

For peer review only - http://bmjopen.bmj.com/site/about/guidelines.xhtml

INTRODUCTION

Cortisol is a marker of hypothalamic-pituitary-adrenal (HPA) axis functioning and follows a diurnal rhythm. Large-scale epidemiological studies have measured salivary cortisol sampled several times during the course of a day to capture its rising levels during the awakening response and the subsequent decline across the day. The mental health and wellbeing consequences of raised cortisol levels are of interest particularly among older people given that they have higher evening cortisol levels[1,2] and greater total cortisol output throughout the day[3] compared with younger people.

Positive mental wellbeing is a multidimensional concept that captures hedonic (e.g. happiness and pain avoidance) and eudemonic (e.g. self-realization and psychologically functioning at potential) aspects of mental health that are distinct from depressive illness.[4,5] Cortisol may be linked to a positive psychological state through its effect on mood-altering neurotransmitters including serotonin.[6] Cortisol also has energy-mobilising properties that may in turn promote mental wellbeing.[7]

Two studies of healthy older people found no association between cortisol levels and positive wellbeing (captured by the positive items of the General Health Questionnaire[8] or by daily positive emotions[9]). A study of women aged 65 and over found that greater eudaimonic wellbeing was associated with flatter cortisol slope over the day, though this was due to lower morning cortisol levels which remained low across the day.[10] All studies were small (less than 200 participants). In younger adults (i.e. 55 years and younger), some studies have found that greater positive affect or happiness was associated with lower total cortisol output[11-14] but others have not.[15,16] These studies have not examined prospective

For peer review only - http://bmjopen.bmj.com/site/about/guidelines.xhtml

associations between baseline cortisol patterns and wellbeing at follow-up in large, population-based samples.

The aim of the current study was to draw on individual participant data from four large British cohort studies using meta-analysis to examine the longitudinal associations between diurnal cortisol and positive mental wellbeing captured by an instrument which summarizes positive thoughts and feelings from both hedonic and eudemonic perspectives. We hypothesized that cortisol patterns indicative of disrupted HPA axis functioning (i.e. lower early morning levels, higher evening levels and less steep decline (i.e. flatter response) across the day) would be associated with lower wellbeing at follow-up.

METHODS

Cohort studies

We used the four cohort studies from the Healthy Ageing across the Life Course (HALCyon) cross cohort research programme[17] with data on both cortisol and Warwick Edinburgh Mental Wellbeing Scale: the Caerphilly Prospective Study (CaPS)[18]; the Hertfordshire Cohort Study (HCS)[19]; the MRC National Survey of Health and Development (NSHD)[20]; and the National Child Development Study (NCDS).[21]

Caerphilly Prospective Study (CaPS)

CaPS is a cohort of men who were recruited when they were aged 45–59 years, between 1979 and 1983, from the town of Caerphilly and adjacent villages in South Wales. In the second wave (1984 – 1988) the original cohort was supplemented with men of a similar age who had moved into the defined area. Salivary cortisol was assessed at phase 5 of data collection in

For peer review only - http://bmjopen.bmj.com/site/about/guidelines.xhtml

For peer review only

2000–2004 when participants were aged 65 to 82 years and wellbeing was assessed in 2011 when they were aged 73 to 90 years.

Hertfordshire Cohort Study (HCS)

HCS is a cohort of men and women born in East, North or West Hertfordshire between 1931 and 1939 whose birth and infant records were available and who were alive and still living in Hertfordshire in the 1990s. Cortisol was assessed at wave 1 of the data collection (1999–2004) when participants were aged 60 – 73 years and wellbeing was assessed in 2008 when they were aged 69 – 78.

The MRC National Survey of Health and Development (NSHD)

NSHD is a nationally representative sample of people born in England, Scotland, and Wales during one week in March 1946 followed prospectively since birth. Cortisol and wellbeing data were collected in 2008-10 when cohort members were aged 62-64 years.

The National Child Development Study (NCDS)

NCDS is a nationally representative sample of people born in England, Scotland, and Wales during 1 week in March 1958 followed prospectively since birth. Cortisol was assessed as part of a bio-medical survey (2002–2004) when cohort members were aged 44-45 and wellbeing was assessed at a follow-up (2008-2009) at mean age of 50.7.

Cortisol

In HCS, just one fasting morning serum cortisol sample was ascertained from each participant, frozen and subsequently measured by radioimmunoassay. Salivary cortisol samples were

For peer review only - http://bmjopen.bmj.com/site/about/guidelines.xhtml

collected in CaPS, NSHD and NCDS at multiple times across the day with participants shown how to collect saliva using plain cotton wool swabs (salivettes) at home. Subjects were asked to chew on the salivettes for 1-2 min and a saliva sample was obtained. In CaPS, participants were requested to take samples on waking, 30 minutes later, at 2 pm and 10 pm on two consecutive days. In NSHD, samples were taken on waking, 30 minutes later and at 9 pm. Samples in NCDS were taken in the first 45 minutes after waking and 3 hours later. Samples from CaPS, NSHD and NCDS were frozen and subsequently assayed by radioimmunoassay done at the University of Dresden which specializes in high through-put cortisol assays.[22]

Mental wellbeing

Positive mental wellbeing was assessed using the Warwick Edinburgh Mental Wellbeing Scale (WEMWBS) in all four studies. This self-completion scale captures positive affect, satisfying interpersonal relationships and positive functioning. Items are worded positively and respondents are asked to indicate how frequently, on a five point scale, they have experienced each statement over the last two weeks. Statements include "I've been feeling good about myself", "I've been feeling close to other people", "I've been interested in new things" and "I've been feeling optimistic about the future". Scores theoretically range from 14 to 70 with 70 indicating highest wellbeing. Where three or fewer items were missing, values were imputed based on the average score for completed items (CaPS n=43; HCS n=52; NSHD n=84; NCDS n=103). Internal consistency of the scale in all four cohorts was high (Cronbach alpha=0.91 in HCS, NSHD and NCDS and 0.93 in CaPS.[23] Validation work indicates good construct validity for a single factor structure as well as good criterion validity and test-retest reliability and supports its use in general population samples.[24]

For peer review only - http://bmjopen.bmj.com/site/about/guidelines.xhtml

Covariates

Key covariates that might confound the association between cortisol and wellbeing and which had been assessed in each of the cohorts were chosen *a priori*: sex, age at cortisol measurement, follow-up time to measurement of wellbeing, body mass index (BMI), smoking, and adult socio-economic position.[25-27] The covariates were measured at the same wave as cortisol samples unless otherwise stated. BMI was calculated as measured weight divided by the square of height and was categorized into quartiles when there was evidence of deviation from linearity in the association with wellbeing (NSHD, NCDS). Smoking was reported by participants and dichotomized into current smoker vs ex- and never smoker (NCDS smoking data at age 42 years). Adult socioeconomic position was derived from own occupational class (CaPS at age 47-67, HCS at age 60-73, NSHD at age 53 or earlier if missing, NCDS at age 42) and grouped as manual or non-manual occupation (the latter indicating greater socioeconomic advantage).

Initial treatment of the data and standardization

Cortisol has a marked circadian rhythm and therefore the time of day at which it is sampled will affect its level. In CaPS, NSHD and NCDS actual times when the salivary samples were taken were recorded by participants. Observed values were adjusted for the time of sampling by fitting a linear or polynomial function to the association between cortisol and time of measurement and adding the resulting residuals from the best fit model to the overall mean cortisol value. This gives the estimated cortisol level at the time specified in the protocol for each participant. Morning cortisol (salivary or serum) levels were available in all four cohorts (CaPS, HCS, NSHD and NCDS) and night time values in CaPS and NSHD. For NCDS, the later cortisol measure was taken 3 hours after the morning measure on the same day (around

For peer review only - http://bmjopen.bmj.com/site/about/guidelines.xhtml

11.15AM) and there was no evening measure. However past publications support the notion that the diurnal decline over this shorter period is a good surrogate for the decline from morning till night[28] and hence this measure was used to derive a measure of diurnal slope. HCS collected serum cortisol, the levels of which are around 20 times higher than the free cortisol concentrations found in saliva. However, a study on the relationship between serum and salivary cortisol in healthy individuals[29] showed that correlations were high whether taken at the same time (>0.90) or 70 minutes apart (0.54—0.94). Those participants who reported taking cortico-steroid medication were excluded from the analysis sample (CaPS n=62; HCS n=13; NSHD n=19). In NCDS, participants were excluded from analysis if they reported taking endocrine system medication (n= 396). Salivary cortisol values greater than 100 nmol/L were removed (CaPS n=5; NSHD n=7; NCDS n=21), since high cortisol values can have substantial statistical influence on estimates and it is unclear what these high values represent.[30] Morning salivary cortisol values that were not between 5am and noon were removed and evening values if they were before 8pm, since these participants may be shift-workers with substantially different cortisol profiles.[31] In CaPS, cortisol values were averaged over the same measures obtained on two consecutive days. Early morning salivary cortisol was computed in CaPS and NSHD as the mean of waking and 30 min samples; to be comparable, in NCDS the cortisol measure taken within 45 minutes after waking was used. In HCS morning serum cortisol was used.

To be able to combine the cohorts in a meta-analysis, the cortisol values were standardized by deriving study-specific z-scores. In NCDS, both early and late morning cortisol were positively skewed, as was night time cortisol in CaPS and NSHD, so values were $\log_e$ transformed before they were converted to z-scores. In addition to the early morning and

For peer review only - http://bmjopen.bmj.com/site/about/guidelines.xhtml

night time cortisol measures, we derived the diurnal slope (CaPS, NSHD, NCDS) as early morning value subtracted from the evening (or late morning in NCDS) value and divided by the lapsed time period. The overall slope is negative and so positive z-scores indicate a flatter response.

Statistical Methods

A two stage meta-analysis was performed. In the first stage, we modelled WEMWBS as a function of each diurnal cortisol indicator in turn using linear regression in each cohort separately, with adjustment for i) sex, age at cortisol measurement and follow-up time to the wellbeing measurement, ii) additional adjustment for BMI, smoking status and socioeconomic position. There was no evidence of deviation from linearity in the association between any of the cortisol measures and WEMWBS. There was no evidence of interaction between sex and any of the cortisol measures. In sensitivity analysis to explore possible bias arising from missing covariates , we adjusted for sex and age at cortisol and follow-up time i) using the maximum available sample with cortisol and wellbeing data (results not presented), ii) using the sample restricted to only those participants that had complete data on all covariates. Results did not materially differ for these two samples. In the second stage, cohort-specific estimates were pooled in random-effects meta-analyses[32] chosen a priori due to the expected heterogeneity between the different studies.

Sensitivity analyses

We corrected regression estimates for regression dilution bias arising from error in the measurement of cortisol. The reliability ratios were estimated by regressing the cortisol

For peer review only - http://bmjopen.bmj.com/site/about/guidelines.xhtml

measure on day 2 on the measure on day 1 from CaPS data.[33] This yielded reliability ratios of 0.554, 0.349 and 0.430 for morning, evening and slope cortisol respectively.

RESULTS

The characteristics of the participants of the four cohorts in the analysis are shown in Table 1. Age at time of wellbeing measurement ranged from mean (sd) age of 50.7 (0.1) years in NCDS to 80.1 (3.9) years in CaPS. Mean wellbeing ranged from 49.5 (7.9) in NCDS to 53.3 (10.6) in CaPS and increased with mean age of the cohort. Mean early morning cortisol values were similar for the three cohorts (CaPS, NSHD, NCDS) that had measured salivary cortisol.

We found no evidence of an association between early morning cortisol and wellbeing in the individual cohorts in sex, age and follow-up time adjusted models. The overall pooled estimate was 0.02 (95% CI -0.17,0.21; p=0.8) and there was no evidence of heterogeneity across studies (I^2=2.3; p for heterogeneity=0.4) (Table 2 and Figure 1A). Further adjustment for all covariates did not affect the overall pooled estimate 0.01 (95% CI -0.22,0.24; I^2=18.0; p for heterogeneity=0.3).

Sex, age and follow-up time adjusted associations between evening cortisol and wellbeing in the individual cohorts were in the expected direction (i.e. higher evening cortisol was associated with lower wellbeing) in NSHD:-0.33 (95% CI -0.77,0.11); CaPS -0.98 (95% CI -2.03,0.07). This indicates a weak inverse association between evening cortisol and wellbeing (Table 2 and Figure 1B). Adjustment for body mass index, smoking and social class attenuated this association. Again, there was no evidence of heterogeneity across studies (I^2=19.1; p for heterogeneity =0.3).

For peer review only - http://bmjopen.bmj.com/site/about/guidelines.xhtml

In the pooled analysis a flatter diurnal slope was associated with poorer wellbeing though this was not statistically significant before or after adjustment for all covariates (Table 2 and Figure 1C). In sensitivity analysis, correcting for regression dilution bias, the sex, age and follow-up time adjusted associations between cortisol and wellbeing were 0.036, -1.347 and -0.163 for morning, evening and slope cortisol respectively.

DISCUSSION

Based on meta-analysis of individual participant data from four large cohort studies, we found some evidence that higher evening cortisol was prospectively associated with lower positive mental wellbeing in middle and older age. The magnitude of this association was small (-0.06 standard deviations in wellbeing) in crude analysis though up to -0.17 standard deviations in wellbeing accounting for possible regression dilution bias. This association was attenuated by the inclusion of body mass index, smoking and socioeconomic position. It remains unclear whether obesity is secondary to HPA axis dysregulation so this may represent over-adjustment for a variable on the explanatory pathway, though obesity is not a key determinant of wellbeing. Morning cortisol and diurnal slope were not associated with wellbeing in the current study.

Evening cortisol is arguably the least affected by salivary sampling protocol deviations, which can bias associations between cortisol and wellbeing towards the null.[7] Furthermore, single-day sampling tends to bias cortisol estimates towards state rather than trait values,[30] which may also explain the lack of association with wellbeing up to eight years later. Only one of the included studies captured cortisol profiles on more than one day and this study only on two days. We examined inter-individual differences in cortisol in relation to wellbeing up to

For peer review only - http://bmjopen.bmj.com/site/about/guidelines.xhtml

eight years after assessment of cortisol patterns. It is possible that an association between cortisol and wellbeing would only be evident over a shorter lag time. When measured on the same day, studies have found higher positive affect among those with lower cortisol output.[14,34,35] In addition, lower output in the first 45 minutes after waking[8] and total output across the day[10] has been associated with higher wellbeing over a period of 3-4 weeks. Where intensive study designs have been used to measure intra-individual change based on serial measurements of both cortisol and wellbeing repeated over multiple days, some studies find evidence of an inverse association between cortisol output and positive affect[11,13,36-38] though others do not.[9,15,16] Trait positive affect has been found to predict higher evening cortisol (in men only)[39] though we are not aware that prospective association between baseline cortisol and subsequent mental wellbeing has been assessed. Future studies are warranted in order to examine the longitudinal association between mental wellbeing and cortisol sampled over multiple days to more accurately capture trait cortisol.

Though the evidence remains inconsistent at present, explanatory pathways include the over-activation of the HPA axis which interacts with the serotonin system and may ultimately result in serotonin depletion, increasing proneness to negative emotional states and reducing positive emotionality.[6,40,41] In addition, it has been proposed that the energy-mobilising properties of cortisol may underlie an association with positive mental wellbeing and that this pathway may be less relevant for understanding an association between cortisol and mental ill health.[7] The evidence base is currently too limited to determine whether diurnal cortisol is related to different components of positive mental wellbeing and to negative mental health in the same way though there is some indication that wellbeing and illbeing do not show the same correlations with cortisol slope.[10] The current

For peer review only - http://bmjopen.bmj.com/site/about/guidelines.xhtml

study used the Warwick Edinburgh Mental Wellbeing Scale, designed to capture both hedonic and eudemonic wellbeing over the last two weeks. Future research might explore a wider set of hedonic and eudemonic components of mental wellbeing, as well as measures of mental ill health, in a single analytical sample to establish whether they show the same or different relationships with diurnal cortisol.

Other limitations should be acknowledged. There were differences in the protocol for the collection of cortisol although we did not find evidence of between-study heterogeneity in the estimates from the meta-analysis (with all I^2 values below 21%). We used clock time but cortisol patterns are more closely anchored to waking time. Participants were instructed to provide samples at specified times post-waking but actual waking time was not recorded. In addition, a maximum of four samples per day were collected and additional measures may have provided more accurate assessment of diurnal cortisol, especially diurnal slope.

Nevertheless, the current study also has strengths. It includes a large number of participants (ranging from 1756 to 7515 in each analysis and considerably larger than any previous study), in population-based samples, using harmonized measures of wellbeing and covariates. Individual participant data meta-analysis was used, which has advantages over aggregate meta-analysis including greater statistical power and standardization of the derivation of variables and analytical models.[42]

In summary, the findings from this meta-analysis do not provide support for the hypothesis that cortisol profiles indicative of disrupted HPA axis functioning has strong associations positive mental wellbeing in healthy middle-aged and older people. Of the three diurnal cortisol levels considered here, only evening cortisol showed a prospective association with wellbeing and only in minimally-adjusted analysis. However, cortisol was sampled on only one or two days and studies with samples across multiple days may find stronger

For peer review only - http://bmjopen.bmj.com/site/about/guidelines.xhtml

For peer review only

associations if they better characterize cortisol patterns, though it is likely that any associations, if found, will be of modest to moderate magnitude.

For peer review only

For peer review only - http://bmjopen.bmj.com/site/about/guidelines.xhtml

Contributor statement:
YBS, CC, CG, CP and DK conceptualised the study. MS, YBS, MG, MCG and RC identified the variables, cleaned the data and undertook data analysis. All authors contributed to reviewing and interpreting the results, commenting on the manuscript and approved the final version.

Competing interests:
Authors have no conflicts of interest to report.

Funding:
MS, RC and DK are supported by the UK Medical Research Council (Programme codes MRC_MC_UU_12019/1; MRC_MC_UU_12019/4; MRC_MC_UU_12019/5). The MRC National Survey of Health and Development is funded by the UK Medical Research Council.

HALCyon was funded by the New Dynamics of Ageing (RES-353-25-0001) and MG was supported by this grant.

Data collection of cortisol in NCDS at 45 years was funded by the United Kingdom Medical Research Council, grant G0000934. This research was supported by the National Institute for Health Research Biomedical Research Centre at Great Ormond Street Hospital for Children NHS Foundation Trust and University College London.

The funders had no role in the study design, data collection, data analysis, data interpretation, writing of the report or the decision to submit the article for publication.

Data sharing statement:
MRC National Survey of Health and Development data used in this publication are available to bona fide researchers upon request to the NSHD Data Sharing Committee via a standard application procedure. Further details can be found at http://www.nshd.mrc.ac.uk/data. doi: 10.5522/NSHD/Q101; doi:10.5522/NSHD/Q102.

All requests for collaboration on the Caerphilly Prospective Study are reviewed by an independent steering committee (http://www.bris.ac.uk/social-community-medicine/projects/caerphilly/collaboration/).

National Child Development Study data are available via registration with the UK Data Service.

The Hertfordshire Cohort Study has governance and access arrangements that comply with MRC data sharing policy. The survey data are accessible to bona fide researchers by contacting Professor Cyrus Cooper, Director of the MRC Lifecourse Epidemiology Unit at the University of Southampton, who can forward a collaborators' agreement.

Acknowledgements:
The authors thank Stephanie Black for her contribution to data collation, statistical analysis and interpretation of the results presented here.

The authors are grateful to the Centre for Longitudinal Studies (CLS), UCL Institute of Education for the use of these data and to the UK Data Service for making them available.

For peer review only - http://bmjopen.bmj.com/site/about/guidelines.xhtml

However, neither CLS nor the UK Data Service bear any responsibility for the analysis or interpretation of these data.

For peer review only

For peer review only - http://bmjopen.bmj.com/site/about/guidelines.xhtml

REFERENCES

1. Lupien S, Lecours AR, Schwartz G, et al. Longitudinal study of basal cortisol levels in healthy elderly subjects: evidence for subgroups. *Neurobiol Aging* 1996;17:95–105.

2. Herbert J, Goodyer IM, Grossman, AB, et al, Do corticosteroids damage the brain? *J Neuroendocrinol* 2006;18:393-411.

3. Kumari M, Badrick E, Sacker A, et al.2010a. Identifying patterns in cortisol secretion in an older population. Findings from the Whitehall II study. *Psychoneuroendocrinology* 2010;35:1091-9.

4. Ryff CD, Singer B. The contours of positive human health. *Psychol Inq* 1998;9:1-28.

5. Huppert FA, Whittington JE. Evidence for the independence of positive and negative well-being: implications for quality of life assessment. *Br J Health Psychol* 2003;8:107-22.

6. Braquehais MD, Picouto MD, Casas M, et al. Sher, L., 2012. Hypothalamic-pituitary-adrenal axis dysfunction as a neurobiological correlate of emotion dysregulation in adolescent suicide. World J. Pediatr. 8, 197-206.

7. Smyth N, Thorn L, Hucklebridge F, et al. Post awakening salivary cortisol secretion and trait well-being: The importance of sample timing accuracy. *Psychoneuroendocrinology* 2015;58:141-51.

8. Evans P, Forte D, Jacobs C, et al. Cortisol secretory activity in older people in relation to positive and negative well-being. *Psychoneuroendocrinology* 2007;32:922-930.

9. Adam EK, Hawkley LC, Kudielka BM, et al. Day-to-day dynamics of experience – cortisol associations in a population-based sample of older adults. *Proc Natl Acad Sci USA* 2006;103:17058-63.

For peer review only - http://bmjopen.bmj.com/site/about/guidelines.xhtml

10. Ryff CD, Love GD, Urry HL, et al. Psychological well-being and ill-being: do they have distinct or mirrored biological correlates? *Psychother Psychosom* 2006;75:85–95.

11. Smyth J, Ockenfels MC, Porter L, et al. Stressors and mood measured on a momentary basis are associated with salivary cortisol secretion. *Psychoneuroendocrinology* 1998;23:353–370.

12. Lai JC, Evans PD, Ng SH, et al. Optimism, positive affectivity, and salivary cortisol. *Br J Health Psychol* 2005;10:467-84.

13. Steptoe A, Wardle J. Positive affect and biological function in everyday life. *Neurobiol Aging* 2005;26 Suppl 1:108-12.

14. Steptoe A, Wardle J, Marmot M, et al. Positive affect and health-related neuroendocrine, cardiovascular, and inflammatory processes. *Proc Natl Acad Sci USA* 2005;102(18):6508-12.

15. van Eck M, Berkhof H, Nicolson N, et al. The effects of perceived stress, traits, mood states, and stressful daily events on salivary cortisol. *Psychosom Med* 1996;58:447-58.

16. Jacobs N, Myin-Germeys I, Derom C, et al. A momentary assessment study of the relationship between affective and adrenocortical stress responses in daily life. *Biol Psychol* 2007;74:60–66.

17. Kuh D, Cooper R, Richards M, et al. A life course approach to healthy ageing: the HALCyon programme. *Public Health* 2012;126:193-5.

18. The Caerphilly and Speedwell Collaborative Group. Caerphilly and Speedwell collaborative heart disease studies. *J Epidemiol Community Health* 1984;38:259–262.

19. Syddall HE, Aihie SA, Dennison EM, et al. Cohort profile: the Hertfordshire cohort study. *Int J Epidemiol* 2005;34:1234—1242.

For peer review only - http://bmjopen.bmj.com/site/about/guidelines.xhtml

20. Kuh D, Pierce M, Adams J, et al. Cohort Profile: updating the cohort profile for the MRC National Survey of Health and Development: a new clinic-based data collection for ageing research. *Int J Epidemiol* 2011;40:e1—e9.

21. Power C, Elliott J. Cohort profile: 1958 British birth cohort (National Child Development Study). *Int J Epidemiol* 2006;35:34-41.

22. Kirschbaum C, Hellhammer DH. Salivary cortisol in psychobiological research: an overview. *Neuropsychobiology* 1989;22:150—169.

23. Gale CR, Cooper R, Craig L, et al. Cognitive function in childhood and lifetime cognitive change in relation to mental wellbeing in four cohorts of older people. PLoS One 2012;7(9):e44860.

24. Tennant R, Hiller L, Fishwick R, et al. The Warwick-Edinburgh Mental Well-being Scale (WEMWBS): development and UK validation. *Health Qual. Life Outcomes* 2007;27(5):63.

25. Badrick E, Bobak M, Britton A, et al. The relationship between alcohol consumption and cortisol secretion in an aging cohort. *J Clin Endocrinol Metab* 2008;93:750–757.

26. Kumari M, Badrick E, Chandola T, et al. Measures of social position and cortisol secretion in an aging population: findings from the Whitehall II study. *Psychosom Med* 2010;72:27-34.

27. Geoffroy MC, Li L, Power C. Depressive symptoms and body mass index: co-morbidity and direction of association in a British birth cohort followed over 50 years. *Psychol Med* 2014;44:2641-2652.

28. Power C, Li L, Hertzman C. Cognitive development and cortisol patterns in mid-life: findings from a British birth cohort. *Psychoneuroendocrinology* 2008;33:530-9.

29. Reid JD, Intrieri RC, Susman EJ, et al. The relationship of serum and salivary cortisol in a sample of healthy elderly. *J Gerontol* 1992;47:176—179.

For peer review only - http://bmjopen.bmj.com/site/about/guidelines.xhtml

30. Adam EK, Kumari M. Assessing salivary cortisol in large-scale, epidemiological research. *Psychoneuroendocrinology* 2009;34:1423-36.

31. Bostock S, Steptoe A. Influences of early shift work on the diurnal cortisol rhythm, mood and sleep: Within-subject variation in male airline pilots. *Psychoneuroendocrinology* 2013;38:533–541

32. DerSimonian R, Laird N. Meta-analysis in clinical trials. *Control Clin Trials* 1986;7:177-88.

33. Berglund L. Regression dilution bias: Tools for correction methods and sample size calculation. *Upsala J Med Sci* 2012;117:279-83.

34. Steptoe A, Gibson EL, Hamer M, et al. Neuroendocrine and cardiovascular correlates of positive affect measured by ecological momentary assessment and by questionnaire. *Psychoneuroendocrinology* 2007;32:56-64.

35. Steptoe A, O'Donnell K, Badrick E, et al. Neuroendocrine and inflammatory factors associated with positive affect in healthy men and women: the Whitehall II study. *Am J Epidemiol* 2008;167:96-102.

36. Hoppmann CA, Klumb PL. Daily goal pursuits predict cortisol secretion and mood states in employed parents with preschool children. *Psychosom Med* 2006;68:887-94.

37. Davydov DM, Shapiro D, Goldstein IB, et al. Moods in everyday situations: effects of menstrual cycle, work, and stress hormones. *J Psychosom Res* 2005;58:343–349.

38. Simpson EE, McConville C, Rae, G., et al. Salivary cortisol, stress and mood in healthy older adults: the Zenith study. *Biol Psychol* 2008;78(1):1–9

39. Polk DE, Cohen S, Doyle WJ, et al. State and trait affect as predictors of salivary cortisol in healthy adults. *Psychoneuroendocrinology* 2005;30:261–272.

For peer review only - http://bmjopen.bmj.com/site/about/guidelines.xhtml

40. Dinan TG. Glucocorticoids and the genesis of depressive illness. A psychobiological model. *Br J Psych* 1994;164:365-71.

41. Stokes PE. The potential role of excessive cortisol induced by HPA hyperfunction in the pathogenesis of depression. *Eur Neuropsychopharm* 1995;77-82.

42. Riley RD, Lambert PC, Abo-Zaid G. Meta-analysis of individual participant data: rationale, conduct, and reporting. *Br Med J* 2010;340, 221.

For peer review only

Table 1: Descriptive statistics of participants by study

	CaPS	HCS	NSHD	NCDS
Sample with WEMWBS and cortisol; n	592	1055	1736	5337
Gender; n(%) male	530 (100)	463 (44)	809 (47)	2464 (50)
Age range at cortisol measurement (years)	70 - 82	60 - 73	62 – 64	44 - 46
Age range at WEMWBS assessment (years); [mean (sd)]	73 - 90 [80.1 (3.9)]	69 – 78 [73.2 (2.4)]	62 - 64 [63.6 (0.8)]	50 - 51 [50.7(0.1)]
BMI (kg/m^2); mean(sd)	28.2 (3.8)	26.9 (4.2)	27.9 (4.8)	27.1 (4.7)
Adult current smoking status; n (%) smoker	53 (11.0)	84 (8.1)	160 (10.2)	893 (18.5)
Adult social class; n(%) manual	303 (59.5)	400 (39)	499 (29.2)	1626 (34.1)
WEMWBS score; mean (sd)	53.3 (10.6)	51.7 (8.1)	51.8 (8.0)	49.5 (7.9)
Sampling times; mean				
Waking sample	7.37AM	N/A	7.11AM	N/A
Waking + 30mins (+45 mins for NCDS)	8.13AM		7.42AM	8.11AM
Waking + 3h 45 mins	N/A		N/A	11:11AM
2pm sample	2.11PM		N/A	N/A
Evening sample	10.05PM		9.27PM	N/A
Serum cortisol (nmol/L)	N/A	258.1 (81.2)	N/A	N/A
Salivary cortisol (nmol/L)				
Early morning; mean (sd)	19.7 (9.5)		22.9 (9.6)	21.3 (10.8)
Night time; median (IQR)	2.3 (1.5, 3.5)		2.4 (1.7,3.7)	N/A
Diurnal slope (nmol/L/h); mean (sd)	-1.13 (0.7)		-1.43 (0.7)	-4.3 (3.8)
Cortisol Awakening Response; mean (sd)	2.3 (9.4)		6.4 (11.7)	N/A
Exclusions due to being on corticosteroids; n(%)	62 (10%)	13 (1.2%)	19(1.1%)	396 (7.5)[a]

[a]endocrine medication

For peer review only

Table 2. Overall summary estimates of effect for the associations between cortisol measures and wellbeing from a series of meta-analyses.

	Included cohorts	Number of individuals	Mean difference in WEMWBS score (95% CI) per SD increase in cortisol							
			Model 1[a]				Model 2[b]			
			**Regression coefficient (95% CI)**	P-value	**Tests of heterogeneity I^2(%)**	P-value[c]	**Regression coefficient (95% CI)**	P-value	**Tests of heterogeneity I^2(%)**	P-value[c]
Early morning cortisol	All	7515	0.02 (-0.17,0.21)	0.8	2.3	0.4	0.01 (-0.22,0.24)	0.9	18.0	0.3
Evening cortisol	CaPS NSHD	1756	-0.47 (-1.00,0.05)	0.08	20.3	0.3	-0.31 (-0.83,0.21)	0.2	19.1	0.3
Flatter diurnal slope	CaPS NSHD NCDS	6490	-0.07 (-0.27,0.14)	0.5	0.0	0.6	-0.08 (-0.28,0.13)	0.5	0.0	0.6

[a]sex, age at cortisol assessment, follow-up time to wellbeing assessment; [b]Model 1 plus body mass index, smoking status, adult social class; [c]p-values from Cochran's Q statistic performed as a test of between-study heterogeneity

For peer review only - http://bmjopen.bmj.com/site/about/guidelines.xhtml

For peer review only

Figure 1. Meta-analysis of the association between wellbeing and (A) early morning cortisol; (B) evening cortisol; (C) flatter diurnal slope. All estimates are adjusted for sex and follow-up time.

For peer review only

For peer review only - http://bmjopen.bmj.com/site/about/guidelines.xhtml

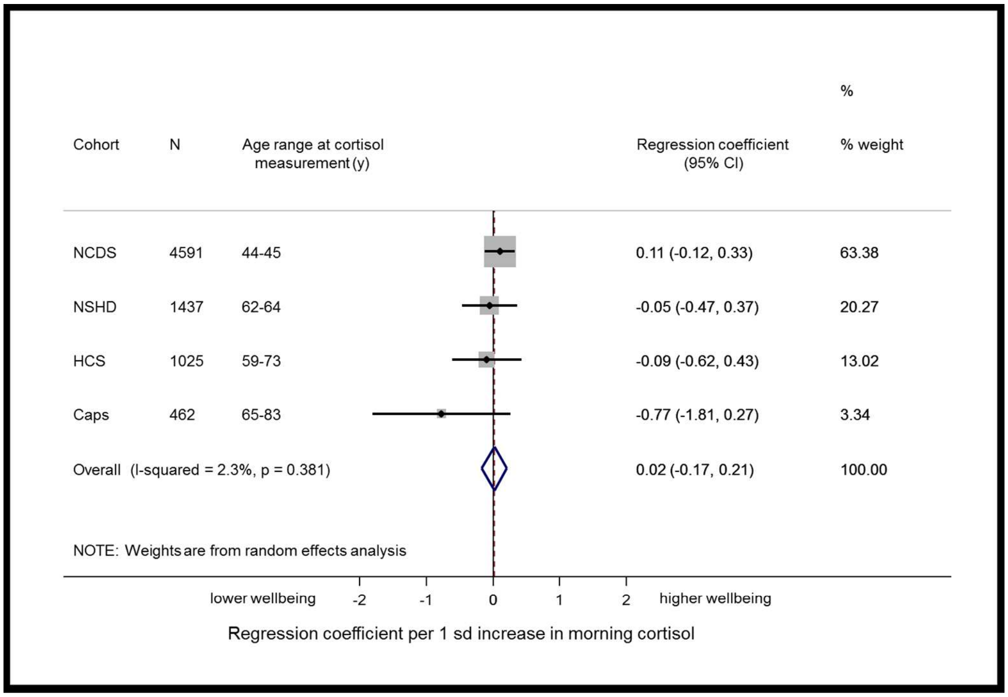

Figure 1A

eview only

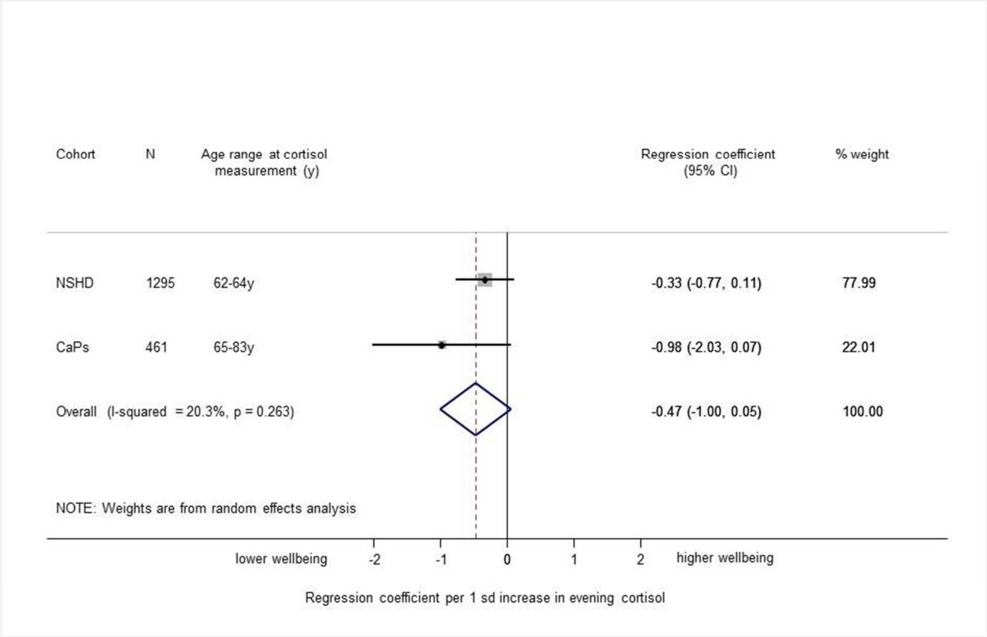

Figure 1B

254x190mm (96 x 96 DPI)

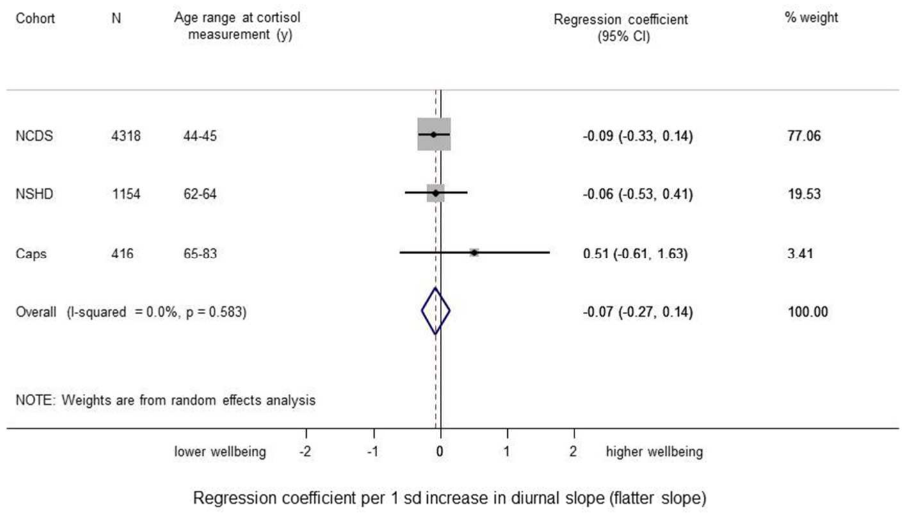

Figure 1C

254x190mm (96 x 96 DPI)

STROBE Statement—checklist of items that should be included in reports of observational studies

	Recommendation	Page
**Title and abstract**	(*a*) Indicate the study's design with a commonly used term in the title or the abstract	1,2
	(*b*) Provide in the abstract an informative and balanced summary of what was done and what was found	1,2
**Introduction**		
Background/rationale	Explain the scientific background and rationale for the investigation being reported	4
Objectives	State specific objectives, including any prespecified hypotheses	5
**Methods**		
Study design	Present key elements of study design early in the paper	5
Setting	Describe the setting, locations, and relevant dates, including periods of recruitment, exposure, follow-up, and data collection	5,6
Participants	(*a*) *Cohort study*—Give the eligibility criteria, and the sources and methods of selection of participants. Describe methods of follow-up	5,6
	(*b*) *Cohort study*—For matched studies, give matching criteria and number of exposed and unexposed	N/A
Variables	Clearly define all outcomes, exposures, predictors, potential confounders, and effect modifiers. Give diagnostic criteria, if applicable	6-8
Data sources/ measurement	For each variable of interest, give sources of data and details of methods of assessment (measurement). Describe comparability of assessment methods if there is more than one group	5-7
Bias	Describe any efforts to address potential sources of bias	10
Study size	Explain how the study size was arrived at	9, Table 1
Quantitative variables	Explain how quantitative variables were handled in the analyses. If applicable, describe which groupings were chosen and why	10
Statistical methods	(*a*) Describe all statistical methods, including those used to control for confounding	10
	(*b*) Describe any methods used to examine subgroups and interactions	10
	(*c*) Explain how missing data were addressed	10
	(*d*) *Cohort study*—If applicable, explain how loss to follow-up was addressed	
	(*e*) Describe any sensitivity analyses	10-11

Continued on next page

For peer review only

**Results**		**Page**
Participants	(a) Report numbers of individuals at each stage of study—eg numbers potentially eligible, examined for eligibility, confirmed eligible, included in the study, completing follow-up, and analysed	9, Table 1
	(b) Give reasons for non-participation at each stage	
	(c) Consider use of a flow diagram	N/A
Descriptive data	(a) Give characteristics of study participants (eg demographic, clinical, social) and information on exposures and potential confounders	Table 1
	(b) Indicate number of participants with missing data for each variable of interest	
	(c) *Cohort study*—Summarise follow-up time (eg, average and total amount)	
Outcome data	*Cohort study*—Report numbers of outcome events or summary measures over time	Table 1
Main results	(*a*) Give unadjusted estimates and, if applicable, confounder-adjusted estimates and their precision (eg, 95% confidence interval). Make clear which confounders were adjusted for and why they were included	Table 2
	(*b*) Report category boundaries when continuous variables were categorized	
	(*c*) If relevant, consider translating estimates of relative risk into absolute risk for a meaningful time period	
Other analyses	Report other analyses done—eg analyses of subgroups and interactions, and sensitivity analyses	12
**Discussion**		
Key results	Summarise key results with reference to study objectives	13
Limitations	Discuss limitations of the study, taking into account sources of potential bias or imprecision. Discuss both direction and magnitude of any potential bias	14
Interpretation	Give a cautious overall interpretation of results considering objectives, limitations, multiplicity of analyses, results from similar studies, and other relevant evidence	13-14
Generalisability	Discuss the generalisability (external validity) of the study results	13
**Other information**		
Funding	Give the source of funding and the role of the funders for the present study and, if applicable, for the original study on which the present article is based	24

.

For peer review only - http://bmjopen.bmj.com/site/about/guidelines.xhtml

BMJ Open

Diurnal cortisol and mental wellbeing in middle and older age: evidence from four cohort studies

Journal:	*BMJ Open*
Manuscript ID	bmjopen-2017-016085.R1
Article Type:	Research
Date Submitted by the Author:	09-May-2017
Complete List of Authors:	Stafford, Mai; University College London, MRC Unit for Lifelong Health and Ageing<br>Ben-Shlomo, Yoav; University of Bristol,<br>Cooper, Cyrus; University of Southampton and Southampton University Hospitals NHS Trust, MRC Lifecourse Epidemiology Unit<br>Gale, Catharine; MRC Epidemiology Resource Centre; University of Edinburgh, Department of Psychology<br>Gardner, Mike; University of Oxford, Nuffield Department of Population Health<br>Geoffroy, Marie-Claude; McGill University, McGill Group for Suicide Studies at Douglas Mental Health University Institute; McGill University, Department of Psychiatry<br>Power, Christine<br>Kuh, Diana; MRC Unit for Lifelong Health and Ageing at UCL,<br>Cooper, Rachel; UCL, MRC Unit for Lifelong Health and Ageing at UCL
**Primary Subject Heading**:	Epidemiology
Secondary Subject Heading:	Mental health
Keywords:	meta-analysis, individual participant data, positive psychology

SCHOLARONE™
Manuscripts

Diurnal cortisol and mental wellbeing in middle and older age: evidence from four cohort studies

Authors information

Mai Stafford[1], Yoav Ben-Shlomo[2], Cyrus Cooper[3], Catharine Gale[3,4], Michael P. Gardner[5], Marie-Claude Geoffroy[6], Chris Power[7], Diana Kuh[1], Rachel Cooper[1]

[1]MRC Unit for Lifelong Health and Ageing at UCL, UCL, London, UK

[2]School of Social and Community Medicine, University of Bristol, Bristol, UK

[3]MRC Lifecourse Epidemiology Unit, University of Southampton, Southampton, UK

[4]Department of Psychology, University of Edinburgh, Edinburgh, UK

[5]Nuffield Department of Population Health, University of Oxford, Oxford, UK

[6]McGill Group for Suicide Studies at Douglas Mental Health University Institute; and Department of Psychiatry, McGill University, Montréal, Canada

[7]Population, Policy and Practice Programme, UCL Great Ormond Street Institute of Child Health, UCL, London, UK

Correspondence to: Dr Mai Stafford, MRC Unit for Lifelong Health and Ageing at UCL, 33 Bedford Place, London, WC1B 5JU; email: m.stafford@ucl.ac.uk; tel: +44 207 570 5712

Word count: 3313

Number of figures and tables: 3 figures, 2 tables

For peer review only

For peer review only - http://bmjopen.bmj.com/site/about/guidelines.xhtml

Diurnal cortisol and mental wellbeing in middle and older age: evidence from four cohort studies

ABSTRACT

Objectives: We conducted individual participant meta-analysis to test the hypothesis that cortisol patterns indicative of dysregulated HPA-axis functioning would be prospectively associated with poorer wellbeing at follow-up.

Setting: Four large UK-based cohort studies

Participants: Those providing valid salivary or serum cortisol samples (n=7515 for morning cortisol; n=1756 for evening cortisol) at baseline (age 44-82) and wellbeing data on the Warwick Edinburgh Mental Wellbeing Scale at follow-up (0-8 years) were included.

Results: Wellbeing was not associated with morning cortisol or diurnal slope though a borderline association with evening cortisol was found. Adjusting for sex and follow-up time, each 1 standard deviation increase in evening cortisol was associated with a -0.47 (95% CI -1.00, 0.05) point lower WEMWBS. This was attenuated by adjustment for body mass index, smoking and socioeconomic position. Between-study heterogeneity was low.

Conclusions: This study does not support the hypothesis that diurnal cortisol is prospectively associated with wellbeing up to 8 years later. However, replication in prospective studies with cortisol samples over multiple days is required.

Keywords: meta-analysis; individual participant data; positive psychology;

For peer review only - http://bmjopen.bmj.com/site/about/guidelines.xhtml

Strengths

- Individual participant meta-analysis based on 1612-7515 participants with cortisol data
- Prospective study with up to 8 years of follow-up
- Validated mental wellbeing instrument based on 14 items capturing hedonic and eudaimonic components harmonized across cohorts

Limitations

- Salivary cortisol samples taken over 1 or 2 days; up to a maximum of 4 times per day
- Actual waking time was not recorded in all studies

For peer review only - http://bmjopen.bmj.com/site/about/guidelines.xhtml

INTRODUCTION

Cortisol is a marker of hypothalamic-pituitary-adrenal (HPA) axis functioning and follows a diurnal rhythm. Large-scale epidemiological studies have measured salivary cortisol sampled several times during the course of a day to capture its rising levels during the awakening response and the subsequent decline across the day. The mental health and wellbeing consequences of raised cortisol levels are of interest particularly among older people given that some studies find they have higher evening cortisol levels[1] and greater total cortisol output throughout the day[2] compared with younger people though there is inter-individual variation in age-related change in cortisol [3] and this may reflect age-related change in disease rather than normal ageing [4].

Positive mental wellbeing is a multidimensional concept that captures hedonic (e.g. happiness and experiencing pleasure) and eudemonic (e.g. self-realization and psychologically functioning at potential) aspects of mental health that are distinct from depressive illness.[5] Cortisol may be linked to a positive psychological state through its effect on mood-altering neurotransmitters including serotonin.[6] Cortisol also has energy-mobilising properties that may in turn promote mental wellbeing.[7]

Two studies of healthy older people found no association between cortisol levels and positive wellbeing (captured by the positive items of the General Health Questionnaire[8] or by daily positive emotions[9]). A study of women aged 65 and over found that greater eudaimonic wellbeing was associated with flatter cortisol slope over the day, though this was due to lower morning cortisol levels which remained low across the day.[10] All studies were small (less than 200 participants). In younger adults (i.e. 55 years and younger), some studies

For peer review only - http://bmjopen.bmj.com/site/about/guidelines.xhtml

have found that greater positive affect or happiness was associated with lower total cortisol output[11-14] but others have not.[15,16] These studies have not examined prospective associations between baseline cortisol patterns and wellbeing at follow-up in large, population-based samples. Although stressors are typically associated with HPA axis activation and decreases in vagal tone, some studies suggest that there may in some cases be a subsequent response involving the dorso-vagal parasympathetic system and down-regulation of the HPA axis resulting in low cortisol levels [17]. If this is the case then a long-term inverse association between cortisol and wellbeing may not be evident.

The aim of the current study was to draw on individual participant data from four large British cohort studies using meta-analysis to examine the longitudinal associations between diurnal cortisol and positive mental wellbeing captured by an instrument which summarizes positive thoughts and feelings from both hedonic and eudemonic perspectives. We hypothesized that cortisol patterns indicative of disrupted HPA axis functioning (i.e. lower early morning levels, higher evening levels and less steep decline (i.e. flatter response) across the day) would be associated with lower wellbeing at follow-up.

METHODS

Cohort studies

We used the four cohort studies from the Healthy Ageing across the Life Course (HALCyon) cross cohort research programme[18] with data on both cortisol and Warwick Edinburgh Mental Wellbeing Scale: the Caerphilly Prospective Study (CaPS)[19]; the Hertfordshire Cohort Study (HCS)[20]; the MRC National Survey of Health and Development (NSHD)[21]; and the

For peer review only - http://bmjopen.bmj.com/site/about/guidelines.xhtml

National Child Development Study (NCDS).[22] All studies received appropriate ethical approval.[19-22]

Caerphilly Prospective Study (CaPS)

CaPS is a cohort of men who were recruited when they were aged 45–59 years, between 1979 and 1983, from the town of Caerphilly and adjacent villages in South Wales. In the second wave (1984 – 1988) the original cohort was supplemented with men of a similar age who had moved into the defined area. Salivary cortisol was assessed at phase 5 of data collection in 2000–2004 when participants were aged 65 to 82 years and wellbeing was assessed in 2011 when they were aged 73 to 90 years.

For peer review only

Hertfordshire Cohort Study (HCS)

HCS is a cohort of men and women born in East, North or West Hertfordshire between 1931 and 1939 whose birth and infant records were available and who were alive and still living in Hertfordshire in the 1990s. Cortisol was assessed at wave 1 of the data collection (1999–2004) when participants were aged 60 – 73 years and wellbeing was assessed in 2008 when they were aged 69 – 78.

The MRC National Survey of Health and Development (NSHD)

NSHD is a nationally representative sample of people born in England, Scotland, and Wales during one week in March 1946 followed prospectively since birth. Cortisol and wellbeing data were collected in 2008-10 when cohort members were aged 62-64 years.

The National Child Development Study (NCDS)

For peer review only - http://bmjopen.bmj.com/site/about/guidelines.xhtml

NCDS is a nationally representative sample of people born in England, Scotland, and Wales during 1 week in March 1958 followed prospectively since birth. Cortisol was assessed as part of a bio-medical survey (2002–2004) when cohort members were aged 44-45 and wellbeing was assessed at a follow-up (2008-2009) at mean age of 50.7.

Cortisol

All study members who had not withdrawn or been lost to follow-up at the relevant sweep were invited to participate in the cortisol sampling. In HCS, one fasting morning serum cortisol sample was taken from each participant at a research clinic between 8.30 and 9.30 AM, frozen and subsequently measured by radioimmunoassay. Salivary cortisol samples were collected in CaPS, NSHD and NCDS at multiple times across the day with participants shown how to collect saliva using plain cotton wool swabs (salivettes) at home. Subjects were asked to chew on the salivettes for 1-2 min and a saliva sample was obtained. In CaPS, participants were requested to take samples on waking, 30 minutes later, at 2 pm and 10 pm on two consecutive days. In NSHD, samples were taken on waking, 30 minutes later and at 9 pm. Samples in NCDS were taken in the first 45 minutes after waking and 3 hours later. Samples from CaPS, NSHD and NCDS were frozen and subsequently assayed by radioimmunoassay done at the University of Dresden which specializes in high through-put cortisol assays.[23]

Mental wellbeing

Positive mental wellbeing was assessed using the Warwick Edinburgh Mental Wellbeing Scale (WEMWBS) in all four studies. This self-completion scale captures positive affect, satisfying interpersonal relationships and positive functioning. Items are worded positively and respondents are asked to indicate how frequently, on a five point scale, they have

For peer review only - http://bmjopen.bmj.com/site/about/guidelines.xhtml

For peer review only

experienced each statement over the last two weeks. Statements include "I've been feeling good about myself", "I've been feeling close to other people", "I've been interested in new things" and "I've been feeling optimistic about the future". Scores theoretically range from 14 to 70 with 70 indicating highest wellbeing. Where three or fewer items were missing, values were imputed based on the average score for completed items (CaPS n=43; HCS n=52; NSHD n=84; NCDS n=103). Internal consistency of the scale in all four cohorts was high (Cronbach alpha=0.91 in HCS, NSHD and NCDS and 0.93 in CaPS.[24] Validation work indicates good construct validity for a single factor structure as well as good criterion validity and test-retest reliability and supports its use in general population samples.[25]

Covariates

Key covariates that might confound the association between cortisol and wellbeing and which had been assessed in each of the cohorts were chosen *a priori*: sex, age at cortisol measurement, follow-up time to measurement of wellbeing, body mass index (BMI), smoking, and adult socio-economic position.[26-28] The covariates were measured at the same wave as cortisol samples unless otherwise stated. BMI was calculated as measured weight divided by the square of height and was categorized into quartiles when there was evidence of deviation from linearity in the association with wellbeing (NSHD, NCDS). Smoking was reported by participants and dichotomized into current smoker vs ex- and never smoker (NCDS smoking data at age 42 years). Adult socioeconomic position was derived from own occupational class (CaPS at age 47-67, HCS at age 60-73, NSHD at age 53 or earlier if missing, NCDS at age 42) and grouped as manual or non-manual occupation (the latter indicating greater socioeconomic advantage).

For peer review only - http://bmjopen.bmj.com/site/about/guidelines.xhtml

Initial treatment of the data and standardization

Cortisol has a marked circadian rhythm and therefore the time of day at which it is sampled will affect its level. In CaPS, NSHD and NCDS actual times when the salivary samples were taken were recorded by participants. Observed values were adjusted for the time of sampling by fitting a linear or polynomial function to the association between cortisol and time of measurement and adding the resulting residuals from the best fit model to the overall mean cortisol value. This gives the estimated cortisol level at the time specified in the protocol for each participant. Morning cortisol (salivary or serum) levels were available in all four cohorts (CaPS, HCS, NSHD and NCDS) and night time values in CaPS and NSHD. For NCDS, the later cortisol measure was taken 3 hours after the morning measure on the same day (around 11.15AM) and there was no evening measure. However past publications support the notion that the diurnal decline over this shorter period is a good surrogate for the decline from morning till night[29] and hence this measure was used to derive a measure of diurnal slope. HCS collected serum cortisol, the levels of which are around 20 times higher than the free cortisol concentrations found in saliva. However, a study on the relationship between serum and salivary cortisol in healthy individuals[30] showed that correlations were high whether taken at the same time (>0.90) or 70 minutes apart (0.54—0.94). Those participants who reported taking cortico-steroid medication were excluded from the analysis sample (CaPS n=62; HCS n=13; NSHD n=19). In NCDS, participants were excluded from analysis if they reported taking endocrine system medication (n= 396). Outlying salivary cortisol values greater than 100 nmol/L were removed (CaPS n=5; NSHD n=7; NCDS n=21), since high cortisol values can have substantial statistical influence on estimates and it is unclear what these high values represent.[31] Morning salivary cortisol values that were not between 5am and noon were removed and evening values if they were before 8pm, since these participants with

For peer review only - http://bmjopen.bmj.com/site/about/guidelines.xhtml

atypical sleeping hours may have substantially different cortisol profiles. In CaPS, cortisol values were averaged over the same measures obtained on two consecutive days. Early morning salivary cortisol was computed in CaPS and NSHD as the mean of waking and 30 min samples; to be comparable, in NCDS the cortisol measure taken within 45 minutes after waking was used. In HCS morning serum cortisol was used.

To be able to combine the cohorts in a meta-analysis, the cortisol values were standardized by deriving study-specific z-scores. In NCDS, both early and late morning cortisol were positively skewed, as was night time cortisol in CaPS and NSHD, so values were $\log_e$ transformed before they were converted to z-scores. In addition to the early morning and night time cortisol measures, we derived the diurnal slope (CaPS, NSHD, NCDS) as early morning value subtracted from the evening (or late morning in NCDS) value and divided by the lapsed time period. The overall slope is negative and so positive z-scores indicate a flatter response.

Statistical Methods

A two stage meta-analysis was performed. In the first stage, we modelled WEMWBS as a function of each diurnal cortisol indicator in turn using linear regression in each cohort separately, with adjustment for i) sex, age at cortisol measurement and follow-up time to the wellbeing measurement, ii) additional adjustment for BMI, smoking status and socioeconomic position. There was no evidence of deviation from linearity in the association between any of the cortisol measures and WEMWBS. There was no evidence of interaction between sex and any of the cortisol measures. In sensitivity analysis to explore possible bias arising from missing covariates , we adjusted for sex and age at cortisol and follow-up time i)

For peer review only - http://bmjopen.bmj.com/site/about/guidelines.xhtml

using the maximum available sample with cortisol and wellbeing data (results not presented), ii) using the sample restricted to only those participants that had complete data on all covariates. Results did not materially differ for these two samples. In the second stage, cohort-specific estimates were pooled in random-effects meta-analyses[32] chosen a priori due to the expected heterogeneity between the different studies.

Sensitivity analyses

We corrected regression estimates for regression dilution bias arising from error in the measurement of cortisol. The reliability ratios were estimated by regressing the cortisol measure on day 2 on the measure on day 1 from CaPS data.[33] This yielded reliability ratios of 0.554, 0.349 and 0.430 for morning, evening and slope cortisol respectively.

RESULTS

The characteristics of the participants of the four cohorts in the analysis are shown in Table 1. Age at time of wellbeing measurement ranged from mean (sd) age of 50.7 (0.1) years in NCDS to 80.1 (3.9) years in CaPS. Mean wellbeing ranged from 49.5 (7.9) in NCDS to 53.3 (10.6) in CaPS and increased with mean age of the cohort. Mean early morning cortisol values were similar for the three cohorts (CaPS, NSHD, NCDS) that had measured salivary cortisol. Night time cortisol values were also similar although diurnal slope was more negative (indicating greater decline) in NCDS than in NSHD and CaPS.

We found no evidence of an association between early morning cortisol and wellbeing in the individual cohorts in sex, age and follow-up time adjusted models. The overall pooled estimate was 0.02 (95% CI -0.17,0.21; p=0.8) and there was no evidence of heterogeneity in this

For peer review only - http://bmjopen.bmj.com/site/about/guidelines.xhtml

association across studies (I^2=2.3; p for heterogeneity=0.4) (Table 2 and Figure 1A). Further adjustment for all covariates did not affect the overall pooled estimate 0.01 (95% CI -0.22,0.24; I^2=18.0; p for heterogeneity=0.3).

Sex, age and follow-up time adjusted associations between evening cortisol and wellbeing in the individual cohorts were in the expected direction (i.e. higher evening cortisol was associated with lower wellbeing) in NSHD: -0.33 (95% CI -0.77,0.11); CaPS -0.98 (95% CI -2.03,0.07). This indicates a weak inverse association between evening cortisol and wellbeing (Table 2 and Figure 1B). Adjustment for BMI, smoking and social class attenuated this association. Again, there was no evidence of heterogeneity across studies (I^2=19.1; p for heterogeneity =0.3).

In the pooled analysis a flatter diurnal slope was associated with poorer wellbeing though this was not statistically significant before or after adjustment for all covariates (Table 2 and Figure 1C). Results excluding NCDS (based on decline in cortisol between early and late morning) were similar (0.02 (95% CI -0.41, 0.46)).

In sensitivity analysis, correcting for regression dilution bias, the sex, age and follow-up time adjusted associations between cortisol and wellbeing were 0.036, -1.347 and -0.163 for morning, evening and slope cortisol respectively.

DISCUSSION

Based on meta-analysis of individual participant data from four large cohort studies, we found that morning cortisol and diurnal slope were not associated with wellbeing but

For peer review only - http://bmjopen.bmj.com/site/about/guidelines.xhtml

there was evidence that higher evening cortisol was prospectively associated with lower positive mental wellbeing in middle and older age. The magnitude of this association was small (-0.06 standard deviations in wellbeing) in crude analysis though up to -0.17 standard deviations in wellbeing accounting for possible regression dilution bias. This association was attenuated by the inclusion of BMI, smoking and socioeconomic position. It remains unclear whether obesity is secondary to HPA axis dysregulation so this may represent over-adjustment for a variable on the explanatory pathway. Perceived stress is also linked to lower socioeconomic position, smoking and cortisol levels [27, 30] and so isolating an association between cortisol and wellbeing independently of these factors needs to be interpreted with caution.

Evening cortisol is arguably the least affected by salivary sampling protocol deviations, which can bias associations between cortisol and wellbeing towards the null.[7] Furthermore, single-day sampling tends to bias cortisol estimates towards state rather than trait values,[31] which may also explain the lack of association with wellbeing up to eight years later. Only one of the included studies captured cortisol profiles on more than one day and this study only on two days. We examined inter-individual differences in cortisol in relation to wellbeing up to eight years after assessment of cortisol patterns. It is possible that an association between cortisol and wellbeing would only be evident over a shorter lag time. When measured on the same day, studies have found higher positive affect among those with lower cortisol output.[14,34,35] In addition, lower output in the first 45 minutes after waking[8] and total output across the day[10] has been associated with higher wellbeing over a period of 3-4 weeks. Where intensive study designs have been used to measure intra-individual change based on serial measurements of both cortisol and wellbeing repeated over multiple days, some studies find evidence of an inverse association between cortisol output and positive

For peer review only - http://bmjopen.bmj.com/site/about/guidelines.xhtml

affect[11,13,36-38] though others do not.[9,15,16] Trait positive affect has been found to predict higher evening cortisol (in men only)[39] though we are not aware that a prospective association between baseline cortisol and subsequent mental wellbeing has been assessed. Future studies are warranted in order to examine the longitudinal association between mental wellbeing and cortisol sampled over multiple days to more accurately capture trait cortisol. In addition, cortisol samples taken at regular intervals throughout follow-up would enable us to identify how changes in HPA-axis activation, such as hypocortisolism as a response to chronic stress, might be related to mental wellbeing.

Though the evidence remains inconsistent at present, explanatory pathways include the over-activation of the HPA axis which interacts with the serotonin system and may ultimately result in serotonin depletion, increasing proneness to negative emotional states and reducing positive emotionality.[6,40,41] In addition, it has been proposed that the energy-mobilising properties of cortisol may underlie an association with positive mental wellbeing and that this pathway may be less relevant for understanding an association between cortisol and mental ill health.[7] The evidence base is currently too limited to determine whether diurnal cortisol is related to different components of positive mental wellbeing and to negative mental health in the same way though there is some indication that wellbeing and illbeing do not show the same correlations with cortisol slope.[10] The current study used the Warwick Edinburgh Mental Wellbeing Scale, designed to capture both hedonic and eudemonic wellbeing over the last two weeks. Future research might explore a wider set of hedonic and eudemonic components of mental wellbeing, as well as measures of mental ill health, in a single analytical sample to establish whether they show the same or different relationships with diurnal cortisol.

For peer review only - http://bmjopen.bmj.com/site/about/guidelines.xhtml

Other limitations should be acknowledged. There were differences in the protocol for the collection of cortisol although we did not find evidence of between-study heterogeneity in the estimates from the meta-analysis (with all I^2 values below 21%). We used clock time but cortisol patterns are more closely anchored to waking time. Participants were instructed to provide samples at specified times post-waking but actual waking time was not recorded. In addition, a maximum of four samples per day were collected and additional measures may have provided more accurate assessment of diurnal cortisol, especially diurnal slope. We did not consider factors that may modify the prospective association between cortisol and wellbeing, such as social support which has been shown to buffer the health impact of stress.[42]

Nevertheless, the current study also has strengths. It includes a large number of participants (ranging from 1756 to 7515 in each analysis and considerably larger than any previous study), in population-based samples, using harmonized measures of wellbeing and covariates. Individual participant data meta-analysis was used, which has advantages over aggregate meta-analysis including greater statistical power and standardization of the derivation of variables and analytical models.[43]

In summary, the findings from this meta-analysis do not provide support for the hypothesis that cortisol profiles indicative of disrupted HPA axis functioning has strong associations positive mental wellbeing in healthy middle-aged and older people. Of the three diurnal cortisol levels considered here, only evening cortisol showed a prospective association with wellbeing and only in minimally-adjusted analysis. However, cortisol was sampled on only one or two days and studies with samples across multiple days may find stronger associations if they better characterize cortisol patterns, though it is likely that any associations, if found, will be of modest to moderate magnitude.

For peer review only - http://bmjopen.bmj.com/site/about/guidelines.xhtml

Contributor statement:
YBS, CC, CG, CP and DK conceptualised the study. MS, YBS, MG, MCG and RC identified the variables, cleaned the data and undertook data analysis. All authors contributed to reviewing and interpreting the results, commenting on the manuscript and approved the final version.

Competing interests:
Authors have no conflicts of interest to report.

Funding:
MS, RC and DK are supported by the UK Medical Research Council (Programme codes MRC_MC_UU_12019/1; MRC_MC_UU_12019/4; MRC_MC_UU_12019/5). The MRC National Survey of Health and Development is funded by the UK Medical Research Council.

HALCyon was funded by the New Dynamics of Ageing (RES-353-25-0001) and MG was supported by this grant.

Data collection of cortisol in NCDS at 45 years was funded by the United Kingdom Medical Research Council, grant G0000934. This research was supported by the National Institute for Health Research Biomedical Research Centre at Great Ormond Street Hospital for Children NHS Foundation Trust and University College London.

The funders had no role in the study design, data collection, data analysis, data interpretation, writing of the report or the decision to submit the article for publication.

Data sharing statement:
MRC National Survey of Health and Development data used in this publication are available to bona fide researchers upon request to the NSHD Data Sharing Committee via a standard application procedure. Further details can be found at http://www.nshd.mrc.ac.uk/data. doi: 10.5522/NSHD/Q101; doi:10.5522/NSHD/Q102.

All requests for collaboration on the Caerphilly Prospective Study are reviewed by an independent steering committee (http://www.bris.ac.uk/social-community-medicine/projects/caerphilly/collaboration/).

National Child Development Study data are available via registration with the UK Data Service.

The Hertfordshire Cohort Study has governance and access arrangements that comply with MRC data sharing policy. The survey data are accessible to bona fide researchers by contacting Professor Cyrus Cooper, Director of the MRC Lifecourse Epidemiology Unit at the University of Southampton, who can forward a collaborators' agreement.

Acknowledgements:
The authors thank Stephanie Black for her contribution to data collation, statistical analysis and interpretation of the results presented here.

For peer review only - http://bmjopen.bmj.com/site/about/guidelines.xhtml

The authors are grateful to the Centre for Longitudinal Studies (CLS), UCL Institute of Education for the use of these data and to the UK Data Service for making them available. However, neither CLS nor the UK Data Service bear any responsibility for the analysis or interpretation of these data.

For peer review only

For peer review only - http://bmjopen.bmj.com/site/about/guidelines.xhtml

REFERENCES

1. Herbert J, Goodyer IM, Grossman, AB, et al, Do corticosteroids damage the brain? *J Neuroendocrinol* 2006;18:393-411.

2. Kumari M, Badrick E, Sacker A, et al.2010a. Identifying patterns in cortisol secretion in an older population. Findings from the Whitehall II study. *Psychoneuroendocrinology* 2010;35:1091-9.

3. Lupien S, Lecours AR, Schwartz G, et al. Longitudinal study of basal cortisol levels in healthy elderly subjects: evidence for subgroups. *Neurobiol Aging* 1996;17:95–105.

4. Souza-Talarico, J. N. D., Marin, M. F., Sindi, S., & Lupien, S. J. (2011). Effects of stress hormones on the brain and cognition: evidence from normal to pathological aging. Dementia & Neuropsychologia, 5(1), 8-16.

5. Huppert FA, Whittington JE. Evidence for the independence of positive and negative well-being: implications for quality of life assessment. *Br J Health Psychol* 2003;8:107-22.

6. Braquehais MD, Picouto MD, Casas M, et al. Sher, L., 2012. Hypothalamic-pituitary-adrenal axis dysfunction as a neurobiological correlate of emotion dysregulation in adolescent suicide. World J. Pediatr. 8, 197-206.

7. Smyth N, Thorn L, Hucklebridge F, et al. Post awakening salivary cortisol secretion and trait well-being: The importance of sample timing accuracy. *Psychoneuroendocrinology* 2015;58:141-51.

8. Evans P, Forte D, Jacobs C, et al. Cortisol secretory activity in older people in relation to positive and negative well-being. *Psychoneuroendocrinology* 2007;32:922-930.

For peer review only - http://bmjopen.bmj.com/site/about/guidelines.xhtml

9. Adam EK, Hawkley LC, Kudielka BM, et al. Day-to-day dynamics of experience – cortisol associations in a population-based sample of older adults. *Proc Natl Acad Sci USA* 2006;103:17058-63.

10. Ryff CD, Love GD, Urry HL, et al. Psychological well-being and ill-being: do they have distinct or mirrored biological correlates? *Psychother Psychosom* 2006;75:85–95.

11. Smyth J, Ockenfels MC, Porter L, et al. Stressors and mood measured on a momentary basis are associated with salivary cortisol secretion. *Psychoneuroendocrinology* 1998;23:353–370.

12. Lai JC, Evans PD, Ng SH, et al. Optimism, positive affectivity, and salivary cortisol. *Br J Health Psychol* 2005;10:467-84.

13. Steptoe A, Wardle J. Positive affect and biological function in everyday life. *Neurobiol Aging* 2005;26 Suppl 1:108-12.

14. Steptoe A, Wardle J, Marmot M, et al. Positive affect and health-related neuroendocrine, cardiovascular, and inflammatory processes. *Proc Natl Acad Sci USA* 2005;102(18):6508-12.

15. van Eck M, Berkhof H, Nicolson N, et al. The effects of perceived stress, traits, mood states, and stressful daily events on salivary cortisol. *Psychosom Med* 1996;58:447-58.

16. Jacobs N, Myin-Germeys I, Derom C, et al. A momentary assessment study of the relationship between affective and adrenocortical stress responses in daily life. *Biol Psychol* 2007;74:60–66.

17. Porges SW. The polyvagal theory: New insights into adaptive reactions of the autonomic nervous system. *Cleve Clin J Med* 2009; 76(Suppl 2):S86–S90.

18. Kuh D, Cooper R, Richards M, et al. A life course approach to healthy ageing: the HALCyon programme. *Public Health* 2012;126:193-5.

For peer review only - http://bmjopen.bmj.com/site/about/guidelines.xhtml

19. The Caerphilly and Speedwell Collaborative Group. Caerphilly and Speedwell collaborative heart disease studies. *J Epidemiol Community Health* 1984;38:259–262.

20. Syddall HE, Aihie SA, Dennison EM, et al. Cohort profile: the Hertfordshire cohort study. *Int J Epidemiol* 2005;34:1234—1242.

21. Kuh D, Pierce M, Adams J, et al. Cohort Profile: updating the cohort profile for the MRC National Survey of Health and Development: a new clinic-based data collection for ageing research. *Int J Epidemiol* 2011;40:e1—e9.

22. Power C, Elliott J. Cohort profile: 1958 British birth cohort (National Child Development Study). *Int J Epidemiol* 2006;35:34-41.

23. Kirschbaum C, Hellhammer DH. Salivary cortisol in psychobiological research: an overview. *Neuropsychobiology* 1989;22:150—169.

24. Gale CR, Cooper R, Craig L, et al. Cognitive function in childhood and lifetime cognitive change in relation to mental wellbeing in four cohorts of older people. PLoS One 2012;7(9):e44860.

25. Tennant R, Hiller L, Fishwick R, et al. The Warwick-Edinburgh Mental Well-being Scale (WEMWBS): development and UK validation. *Health Qual. Life Outcomes* 2007;27(5):63.

26. Badrick E, Bobak M, Britton A, et al. The relationship between alcohol consumption and cortisol secretion in an aging cohort. *J Clin Endocrinol Metab* 2008;93:750–757.

27. Kumari M, Badrick E, Chandola T, et al. Measures of social position and cortisol secretion in an aging population: findings from the Whitehall II study. *Psychosom Med* 2010;72:27-34.

For peer review only - http://bmjopen.bmj.com/site/about/guidelines.xhtml

28. Geoffroy MC, Li L, Power C. Depressive symptoms and body mass index: co-morbidity and direction of association in a British birth cohort followed over 50 years. *Psychol Med* 2014;44:2641-2652.

29. Power C, Li L, Hertzman C. Cognitive development and cortisol patterns in mid-life: findings from a British birth cohort. *Psychoneuroendocrinology* 2008;33:530-9.

30. Reid JD, Intrieri RC, Susman EJ, et al. The relationship of serum and salivary cortisol in a sample of healthy elderly. *J Gerontol* 1992;47:176—179.

31. Adam EK, Kumari M. Assessing salivary cortisol in large-scale, epidemiological research. *Psychoneuroendocrinology* 2009;34:1423-36.

32. DerSimonian R, Laird N. Meta-analysis in clinical trials. *Control Clin Trials* 1986;7:177-88.

33. Berglund L. Regression dilution bias: Tools for correction methods and sample size calculation. *Upsala J Med Sci* 2012;117:279-83.

34. Steptoe A, Gibson EL, Hamer M, et al. Neuroendocrine and cardiovascular correlates of positive affect measured by ecological momentary assessment and by questionnaire. *Psychoneuroendocrinology* 2007;32:56-64.

35. Steptoe A, O'Donnell K, Badrick E, et al. Neuroendocrine and inflammatory factors associated with positive affect in healthy men and women: the Whitehall II study. *Am J Epidemiol* 2008;167:96-102.

36. Hoppmann CA, Klumb PL. Daily goal pursuits predict cortisol secretion and mood states in employed parents with preschool children. *Psychosom Med* 2006;68:887-94.

37. Davydov DM, Shapiro D, Goldstein IB, et al. Moods in everyday situations: effects of menstrual cycle, work, and stress hormones. *J Psychosom Res* 2005;58:343–349.

For peer review only - http://bmjopen.bmj.com/site/about/guidelines.xhtml

38. Simpson EE, McConville C, Rae, G., et al. Salivary cortisol, stress and mood in healthy older adults: the Zenith study. *Biol Psychol* 2008;78(1):1–9

39. Polk DE, Cohen S, Doyle WJ, et al. State and trait affect as predictors of salivary cortisol in healthy adults. *Psychoneuroendocrinology* 2005;30:261–272.

40. Dinan TG. Glucocorticoids and the genesis of depressive illness. A psychobiological model. *Br J Psych* 1994;164:365-71.

41. Stokes PE. The potential role of excessive cortisol induced by HPA hyperfunction in the pathogenesis of depression. *Eur Neuropsychopharm* 1995;77-82.

42. Hostinar CE, Gunnar MR. Social Support Can Buffer against Stress and Shape Brain Activity. *AJOB Neurosci* 2015;6:34-42.

43. Riley RD, Lambert PC, Abo-Zaid G. Meta-analysis of individual participant data: rationale, conduct, and reporting. *Br Med J* 2010;340, 221.

review only

For peer review only - http://bmjopen.bmj.com/site/about/guidelines.xhtml

Table 1: Descriptive statistics of participants by study

	CaPS	HCS	NSHD	NCDS
Sample with WEMWBS and cortisol; n	592	1055	1736	5337
Gender; n(%) male	530 (100)	463 (44)	809 (47)	2464 (50)
Age range at cortisol measurement (years); [mean (sd)]	65 – 82 [72.8 (4.0)]	60 – 73 [66.6 (2.7)]	62 – 64 [63.6 (0.8)]	44 – 46 [44.1 (0.2)]
Age range at WEMWBS assessment (years); [mean (sd)]	73 - 90 [80.1 (3.9)]	69 – 78 [73.2 (2.4)]	62 - 64 [63.6 (0.8)]	50 – 51 [50.7(0.1)]
Follow-up time cortisol to WEMWBS assessment (years); mean (sd)	7.6 (0.4)	6.6 (1.2)	0	6.6 (0.3)
BMI (kg/m^2); mean(sd)	28.2 (3.8)	26.9 (4.2)	27.9 (4.8)	27.1 (4.7)
Adult current smoking status; n (%) smoker	53 (11.0)	84 (8.1)	160 (10.2)	893 (18.5)
Adult social class; n(%) manual	303 (59.5)	400 (39)	499 (29.2)	1626 (34.1)
WEMWBS score; mean (sd)	53.3 (10.6)	51.7 (8.1)	51.8 (8.0)	49.5 (7.9)
Sampling times; mean				
Waking sample	7.37AM	N/A	7.11AM	N/A
Waking + 30mins (+45 mins for NCDS)	8.13AM		7.42AM	8.11AM
Waking + 3h 45 mins	N/A		N/A	11:11AM
2pm sample	2.11PM		N/A	N/A
Evening sample	10.05PM		9.27PM	N/A
Serum cortisol (nmol/L)	N/A	258.1 (81.2)	N/A	N/A
Salivary cortisol (nmol/L)				
Early morning; mean (sd)	19.7 (9.5)		22.9 (9.6)	21.3 (10.8)
Night time; median (IQR)	2.3 (1.5, 3.5)		2.4 (1.7,3.7)	N/A
Diurnal slope (nmol/L/h); mean (sd)	-1.13 (0.7)		-1.43 (0.7)	-4.3 (3.8)
Exclusions due to being on corticosteroids; n(%)	62 (10%)	13 (1.2%)	19(1.1%)	396 (7.5)[a]

[a]endocrine medication

For peer review only - http://bmjopen.bmj.com/site/about/guidelines.xhtml

Table 2. Overall summary estimates of effect for the associations between cortisol measures and wellbeing from a series of meta-analyses.

	Included cohorts	Number of individuals	Mean difference in WEMWBS score (95% CI) per SD increase in cortisol							
			Model 1[a]				Model 2[b]			
			**Regression coefficient (95% CI)**	P-value	**Tests of heterogeneity I^2(%)**	P-value[c]	**Regression coefficient (95% CI)**	P-value	**Tests of heterogeneity I^2(%)**	P-value[c]
Early morning cortisol	All	7515	0.02 (-0.17,0.21)	0.8	2.3	0.4	0.01 (-0.22,0.24)	0.9	18.0	0.3
Evening cortisol	CaPS NSHD	1756	-0.47 (-1.00,0.05)	0.08	20.3	0.3	-0.31 (-0.83,0.21)	0.2	19.1	0.3
Flatter diurnal slope	CaPS NSHD NCDS	6490	-0.07 (-0.27,0.14)	0.5	0.0	0.6	-0.08 (-0.28,0.13)	0.5	0.0	0.6

[a]sex, age at cortisol assessment, follow-up time to wellbeing assessment; [b]Model 1 plus body mass index, smoking status, adult social class; [c]p-values from Cochran's Q statistic performed as a test of between-study heterogeneity

For peer review only - http://bmjopen.bmj.com/site/about/guidelines.xhtml

Figure Legend

Figure 1. Meta-analysis of the association between wellbeing and (A) early morning cortisol; (B) evening cortisol; (C) flatter diurnal slope. All estimates are adjusted for sex and follow-up time.

For peer review only

For peer review only - http://bmjopen.bmj.com/site/about/guidelines.xhtml

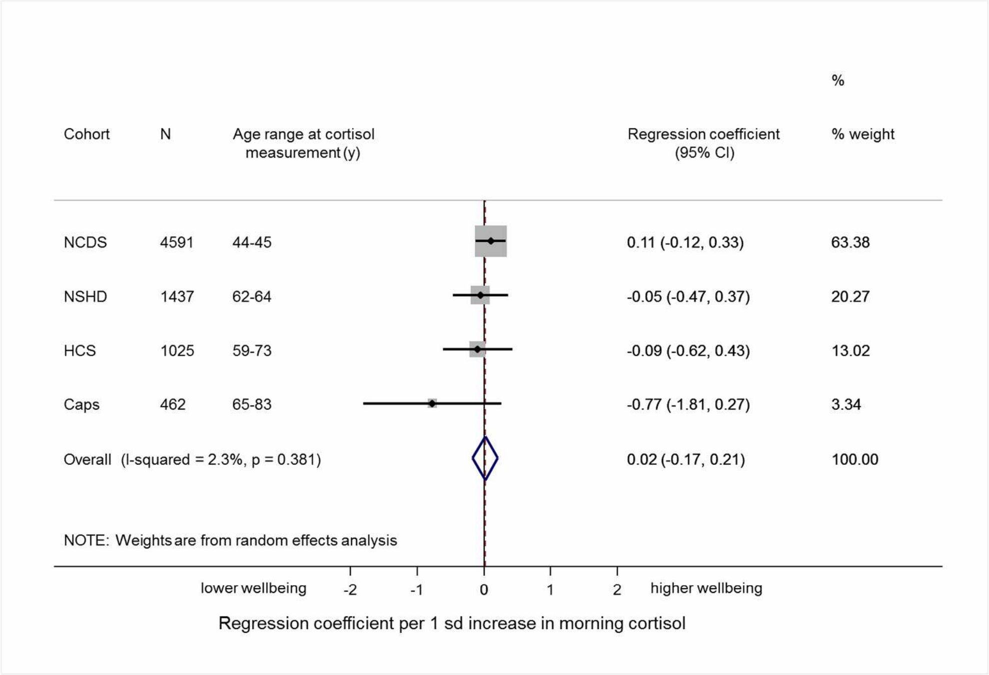

Figure 1A

276x190mm (300 x 300 DPI)

view only

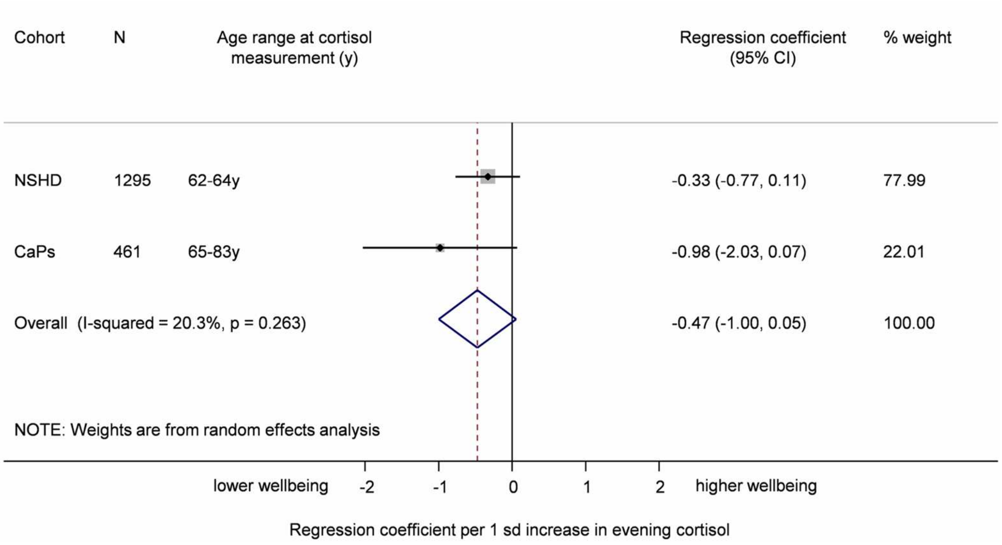

137x74mm (300 x 300 DPI)

review only

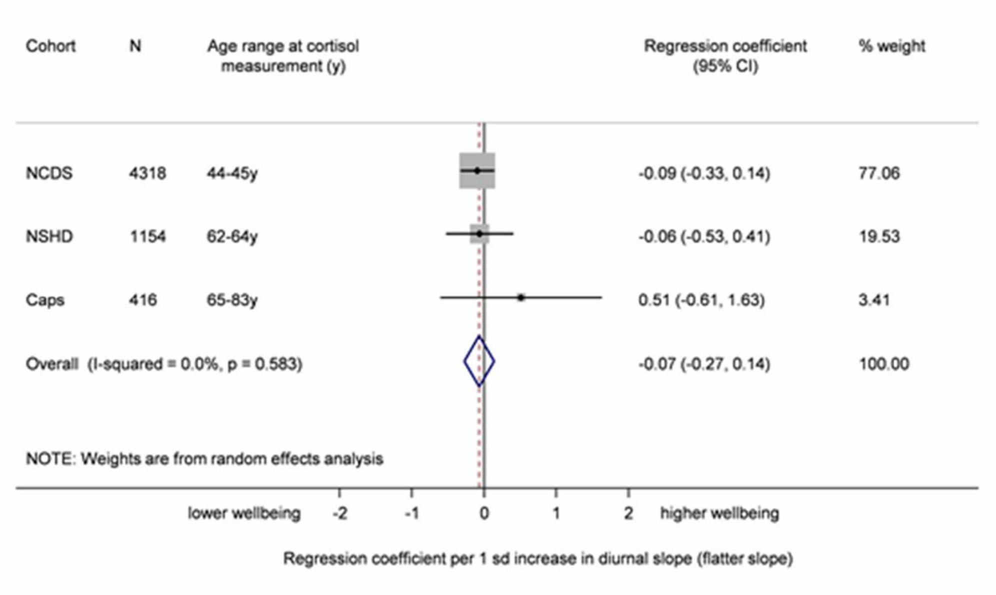

Figure 1C

46x28mm (300 x 300 DPI)

For peer review only - http://bmjopen.bmj.com/site/about/guidelines.xhtml

STROBE Statement—checklist of items that should be included in reports of observational studies

	Recommendation	Page
**Title and abstract**	(*a*) Indicate the study's design with a commonly used term in the title or the abstract	1,2
	(*b*) Provide in the abstract an informative and balanced summary of what was done and what was found	1,2
**Introduction**		
Background/rationale	Explain the scientific background and rationale for the investigation being reported	4
Objectives	State specific objectives, including any prespecified hypotheses	5
**Methods**		
Study design	Present key elements of study design early in the paper	5
Setting	Describe the setting, locations, and relevant dates, including periods of recruitment, exposure, follow-up, and data collection	5,6
Participants	(*a*) *Cohort study*—Give the eligibility criteria, and the sources and methods of selection of participants. Describe methods of follow-up	5,6
	(*b*) *Cohort study*—For matched studies, give matching criteria and number of exposed and unexposed	N/A
Variables	Clearly define all outcomes, exposures, predictors, potential confounders, and effect modifiers. Give diagnostic criteria, if applicable	6-8
Data sources/ measurement	For each variable of interest, give sources of data and details of methods of assessment (measurement). Describe comparability of assessment methods if there is more than one group	5-7
Bias	Describe any efforts to address potential sources of bias	9-10
Study size	Explain how the study size was arrived at	9, Table 1
Quantitative variables	Explain how quantitative variables were handled in the analyses. If applicable, describe which groupings were chosen and why	10
Statistical methods	(*a*) Describe all statistical methods, including those used to control for confounding	10
	(*b*) Describe any methods used to examine subgroups and interactions	10
	(*c*) Explain how missing data were addressed	10
	(*d*) *Cohort study*—If applicable, explain how loss to follow-up was addressed	
	(*e*) Describe any sensitivity analyses	11

Continued on next page

For peer review only

Results		Page
Participants	(a) Report numbers of individuals at each stage of study—eg numbers potentially eligible, examined for eligibility, confirmed eligible, included in the study, completing follow-up, and analysed	9, Table 1
	(b) Give reasons for non-participation at each stage	
	(c) Consider use of a flow diagram	N/A
Descriptive data	(a) Give characteristics of study participants (eg demographic, clinical, social) and information on exposures and potential confounders	Table 1
	(b) Indicate number of participants with missing data for each variable of interest	
	(c) *Cohort study*—Summarise follow-up time (eg, average and total amount)	
Outcome data	*Cohort study*—Report numbers of outcome events or summary measures over time	Table 1
Main results	(*a*) Give unadjusted estimates and, if applicable, confounder-adjusted estimates and their precision (eg, 95% confidence interval). Make clear which confounders were adjusted for and why they were included	Table 2
	(*b*) Report category boundaries when continuous variables were categorized	
	(*c*) If relevant, consider translating estimates of relative risk into absolute risk for a meaningful time period	
Other analyses	Report other analyses done—eg analyses of subgroups and interactions, and sensitivity analyses	12
**Discussion**		
Key results	Summarise key results with reference to study objectives	13
Limitations	Discuss limitations of the study, taking into account sources of potential bias or imprecision. Discuss both direction and magnitude of any potential bias	14
Interpretation	Give a cautious overall interpretation of results considering objectives, limitations, multiplicity of analyses, results from similar studies, and other relevant evidence	13-14
Generalisability	Discuss the generalisability (external validity) of the study results	14
**Other information**		
Funding	Give the source of funding and the role of the funders for the present study and, if applicable, for the original study on which the present article is based	16

.

For peer review only

BMJ Open

Diurnal cortisol and mental wellbeing in middle and older age: evidence from four cohort studies

Journal:	*BMJ Open*
Manuscript ID	bmjopen-2017-016085.R2
Article Type:	Research
Date Submitted by the Author:	28-Jun-2017
Complete List of Authors:	Stafford, Mai; University College London, MRC Unit for Lifelong Health and Ageing<br>Ben-Shlomo, Yoav; University of Bristol,<br>Cooper, Cyrus; University of Southampton and Southampton University Hospitals NHS Trust, MRC Lifecourse Epidemiology Unit<br>Gale, Catharine; MRC Epidemiology Resource Centre; University of Edinburgh, Department of Psychology<br>Gardner, Mike; University of Oxford, Nuffield Department of Population Health<br>Geoffroy, Marie-Claude; McGill University, McGill Group for Suicide Studies at Douglas Mental Health University Institute; McGill University, Department of Psychiatry<br>Power, Christine<br>Kuh, Diana; MRC Unit for Lifelong Health and Ageing at UCL,<br>Cooper, Rachel; UCL, MRC Unit for Lifelong Health and Ageing at UCL
**Primary Subject Heading**:	Epidemiology
Secondary Subject Heading:	Mental health
Keywords:	meta-analysis, individual participant data, positive psychology

SCHOLARONE™
Manuscripts

Diurnal cortisol and mental wellbeing in middle and older age: evidence from four cohort studies

Authors information

Mai Stafford[1], Yoav Ben-Shlomo[2], Cyrus Cooper[3], Catharine Gale[3,4], Michael P. Gardner[5], Marie-Claude Geoffroy[6], Chris Power[7], Diana Kuh[1], Rachel Cooper[1]

[1]MRC Unit for Lifelong Health and Ageing at UCL, UCL, London, UK

[2]School of Social and Community Medicine, University of Bristol, Bristol, UK

[3]MRC Lifecourse Epidemiology Unit, University of Southampton, Southampton, UK

[4]Department of Psychology, University of Edinburgh, Edinburgh, UK

[5]Nuffield Department of Population Health, University of Oxford, Oxford, UK

[6]McGill Group for Suicide Studies at Douglas Mental Health University Institute; and Department of Psychiatry, McGill University, Montréal, Canada

[7]Population, Policy and Practice Programme, UCL Great Ormond Street Institute of Child Health, UCL, London, UK

Correspondence to: Dr Mai Stafford, MRC Unit for Lifelong Health and Ageing at UCL, 33 Bedford Place, London, WC1B 5JU; email: m.stafford@ucl.ac.uk; tel: +44 207 570 5712

Word count: 3313

Number of figures and tables: 4 figures, 2 tables

For peer review only - http://bmjopen.bmj.com/site/about/guidelines.xhtml

For peer review only

Diurnal cortisol and mental wellbeing in middle and older age: evidence from four cohort studies

ABSTRACT

Objectives: We conducted individual participant meta-analysis to test the hypothesis that cortisol patterns indicative of dysregulated HPA-axis functioning would be prospectively associated with poorer wellbeing at follow-up.

Setting: Four large UK-based cohort studies

Participants: Those providing valid salivary or serum cortisol samples (n=7515 for morning cortisol; n=1612 for cortisol awakening response) at baseline (age 44-82) and wellbeing data on the Warwick Edinburgh Mental Wellbeing Scale at follow-up (0-8 years) were included.

Results: Wellbeing was not associated with morning cortisol, diurnal slope or awakening response though a borderline association with evening cortisol was found. Adjusting for sex and follow-up time, each 1 standard deviation increase in evening cortisol was associated with a -0.47 (95% CI -1.00, 0.05) point lower wellbeing. This was attenuated by adjustment for body mass index, smoking and socioeconomic position. Between-study heterogeneity was low.

Conclusions: This study does not support the hypothesis that diurnal cortisol is prospectively associated with wellbeing up to 8 years later. However, replication in prospective studies with cortisol samples over multiple days is required.

Keywords: meta-analysis; individual participant data; positive psychology;

For peer review only

Strengths

- Individual participant meta-analysis based on 1612-7515 participants with cortisol data
- Prospective study with up to 8 years of follow-up
- Validated mental wellbeing instrument based on 14 items capturing hedonic and eudaimonic components harmonized across cohorts

Limitations

- Salivary cortisol samples taken over 1 or 2 days; up to a maximum of 4 times per day
- Actual waking time was not recorded in all studies

For peer review only - http://bmjopen.bmj.com/site/about/guidelines.xhtml

INTRODUCTION

Cortisol is a marker of hypothalamic-pituitary-adrenal (HPA) axis functioning and follows a diurnal rhythm. Large-scale epidemiological studies have measured salivary cortisol sampled several times during the course of a day to capture its rising levels during the awakening response and the subsequent decline across the day. The mental health and wellbeing consequences of raised cortisol levels are of interest particularly among older people given that some studies find they have higher evening cortisol levels[1] and greater total cortisol output throughout the day[2] compared with younger people though there is inter-individual variation in age-related change in cortisol [3] and this may reflect age-related change in disease rather than normal ageing [4].

Positive mental wellbeing is a multidimensional concept that captures hedonic (e.g. happiness and experiencing pleasure) and eudemonic (e.g. self-realization and psychologically functioning at potential) aspects of mental health that are distinct from depressive illness.[5] Cortisol may be linked to a positive psychological state through its effect on mood-altering neurotransmitters including serotonin.[6] Cortisol also has energy-mobilising properties that may in turn promote mental wellbeing.[7]

Two studies of healthy older people found no association between cortisol levels and positive wellbeing (captured by the positive items of the General Health Questionnaire[8] or by daily positive emotions[9]). A study of women aged 65 and over found that greater eudaimonic wellbeing was associated with flatter cortisol slope over the day, though this was due to lower morning cortisol levels which remained low across the day.[10] All studies were small (less than 200 participants). In younger adults (i.e. 55 years and younger), some studies have found that greater positive affect or happiness was associated with lower total cortisol

For peer review only - http://bmjopen.bmj.com/site/about/guidelines.xhtml

output[11-14] but others have not.[15,16] Greater optimism has also been associated with a smaller awakening response.[12]

These studies have not examined prospective associations between baseline cortisol patterns and wellbeing at follow-up in large, population-based samples. Although stressors are typically associated with HPA axis activation and decreases in vagal tone, some studies suggest that there may in some cases be a subsequent response involving the dorso-vagal parasympathetic system and down-regulation of the HPA axis resulting in low cortisol levels [17]. If this is the case then a long-term inverse association between cortisol and wellbeing may not be evident.

The aim of the current study was to draw on individual participant data from four large British cohort studies using meta-analysis to examine the longitudinal associations between diurnal cortisol and positive mental wellbeing captured by an instrument which summarizes positive thoughts and feelings from both hedonic and eudemonic perspectives. We hypothesized that cortisol patterns indicative of disrupted HPA axis functioning (i.e. lower early morning levels, higher evening levels, less steep decline (i.e. flatter response) across the day and larger awakening response) would be associated with lower wellbeing at follow-up.

For peer review only

METHODS

Cohort studies

We used the four cohort studies from the Healthy Ageing across the Life Course (HALCyon) cross cohort research programme[18] with data on both cortisol and Warwick Edinburgh Mental Wellbeing Scale (WEMWBS): the Caerphilly Prospective Study (CaPS)[19]; the Hertfordshire Cohort Study (HCS)[20]; the MRC National Survey of Health and Development

For peer review only - http://bmjopen.bmj.com/site/about/guidelines.xhtml

(NSHD)[21]; and the National Child Development Study (NCDS).[22] All studies received appropriate ethical approval.[19-22]

Caerphilly Prospective Study (CaPS)

CaPS is a cohort of men who were recruited when they were aged 45–59 years, between 1979 and 1983, from the town of Caerphilly and adjacent villages in South Wales. In the second wave (1984 – 1988) the original cohort was supplemented with men of a similar age who had moved into the defined area. Salivary cortisol was assessed at phase 5 of data collection in 2000–2004 when participants were aged 65 to 82 years and wellbeing was assessed in 2011 when they were aged 73 to 90 years.

Hertfordshire Cohort Study (HCS)

HCS is a cohort of men and women born in East, North or West Hertfordshire between 1931 and 1939 whose birth and infant records were available and who were alive and still living in Hertfordshire in the 1990s. Cortisol was assessed at wave 1 of the data collection (1999–2004) when participants were aged 60 – 73 years and wellbeing was assessed in 2008 when they were aged 69 – 78.

The MRC National Survey of Health and Development (NSHD)

NSHD is a nationally representative sample of people born in England, Scotland, and Wales during one week in March 1946 followed prospectively since birth. Cortisol and wellbeing data were collected in 2008-10 when cohort members were aged 62-64 years.

The National Child Development Study (NCDS)

For peer review only - http://bmjopen.bmj.com/site/about/guidelines.xhtml

For peer review only

NCDS is a nationally representative sample of people born in England, Scotland, and Wales during 1 week in March 1958 followed prospectively since birth. Cortisol was assessed as part of a bio-medical survey (2002–2004) when cohort members were aged 44-45 and wellbeing was assessed at a follow-up (2008-2009) at mean age of 50.7.

Cortisol

All study members who had not withdrawn or been lost to follow-up at the relevant sweep were invited to participate in the cortisol sampling. In HCS, one fasting morning serum cortisol sample was taken from each participant at a research clinic between 8.30 and 9.30 AM (the exact timing was not recorded), frozen and subsequently measured by radioimmunoassay. Salivary cortisol samples were collected in CaPS, NSHD and NCDS at multiple times across the day with participants shown how to collect saliva using plain cotton wool swabs (salivettes) at home. Subjects were asked to chew on the salivettes for 1-2 min and a saliva sample was obtained. In CaPS, participants were requested to take samples on waking, 30 minutes later, at 2 pm and 10 pm on two consecutive days. In NSHD, samples were taken on waking, 30 minutes later and at 9 pm. Samples in NCDS were taken in the first 45 minutes after waking and 3 hours later. Samples from CaPS, NSHD and NCDS were frozen and subsequently assayed by radioimmunoassay done at the University of Dresden which specializes in high through-put cortisol assays.[23]

Mental wellbeing

Positive mental wellbeing was assessed using WEMWBS in all four studies. This self-completion scale captures positive affect, satisfying interpersonal relationships and positive functioning. Items are worded positively and respondents are asked to indicate how

For peer review only - http://bmjopen.bmj.com/site/about/guidelines.xhtml

frequently, on a five point scale, they have experienced each statement over the last two weeks. Statements include “I’ve been feeling good about myself”, “I’ve been feeling close to other people”, “I’ve been interested in new things” and “I’ve been feeling optimistic about the future”. Scores theoretically range from 14 to 70 with 70 indicating highest wellbeing. Where three or fewer items were missing, values were imputed based on the average score for completed items (CaPS n=43; HCS n=52; NSHD n=84; NCDS n=103). Internal consistency of the scale in all four cohorts was high (Cronbach alpha=0.91 in HCS, NSHD and NCDS and 0.93 in CaPS.[24] Validation work indicates good construct validity for a single factor structure as well as good criterion validity and test-retest reliability and supports its use in general population samples.[25]

Covariates

Key covariates that might confound the association between cortisol and wellbeing and which had been assessed in each of the cohorts were chosen *a priori*: sex, age at cortisol measurement, follow-up time to measurement of wellbeing, body mass index (BMI), smoking, and adult socio-economic position.[26-28] The covariates were measured at the same wave as cortisol samples unless otherwise stated. BMI was calculated as measured weight divided by the square of height and was categorized into quartiles when there was evidence of deviation from linearity in the association with wellbeing (NSHD, NCDS). Smoking was reported by participants and dichotomized into current smoker vs ex- and never smoker (NCDS smoking data at age 42 years). Adult socioeconomic position was derived from own occupational class (CaPS at age 47-67, HCS at age 60-73, NSHD at age 53 or earlier if missing, NCDS at age 42) and grouped as manual or non-manual occupation (the latter indicating greater socioeconomic advantage).

For peer review only - http://bmjopen.bmj.com/site/about/guidelines.xhtml

Initial treatment of the data and standardization

Cortisol has a marked circadian rhythm and therefore the time of day at which it is sampled will affect its level. In CaPS, NSHD and NCDS actual times when the salivary samples were taken were recorded by participants. Observed values were adjusted for the time of sampling by fitting a linear or polynomial function to the association between cortisol and time of measurement and adding the resulting residuals from the best fit model to the overall mean cortisol value. This gives the estimated cortisol level at the time specified in the protocol for each participant. Morning cortisol (salivary or serum) levels were available in all four cohorts (CaPS, HCS, NSHD and NCDS) and night time values in CaPS and NSHD. For NCDS, the later cortisol measure was taken 3 hours after the morning measure on the same day (around 11.15AM) and there was no evening measure. However past publications support the notion that the diurnal decline over this shorter period is a good surrogate for the decline from morning till night[29] and hence this measure was used to derive a measure of diurnal slope. HCS collected serum cortisol, the levels of which are around 20 times higher than the free cortisol concentrations found in saliva. However, a study on the relationship between serum and salivary cortisol in healthy individuals[30] showed that correlations were high whether taken at the same time (>0.90) or 70 minutes apart (0.54—0.94). Those participants who reported taking cortico-steroid medication were excluded from the analysis sample (CaPS n=62; HCS n=13; NSHD n=19). In NCDS, participants were excluded from analysis if they reported taking endocrine system medication (n= 396). Outlying salivary cortisol values greater than 100 nmol/L were removed (CaPS n=5; NSHD n=7; NCDS n=21), since high cortisol values can have substantial statistical influence on estimates and it is unclear what these high values represent.[31] Morning salivary cortisol values that were not between 5am and noon

For peer review only - http://bmjopen.bmj.com/site/about/guidelines.xhtml

were removed and evening values if they were before 8pm, since these participants with atypical sleeping hours may have substantially different cortisol profiles. In CaPS, cortisol values were averaged over the same measures obtained on two consecutive days. Early morning salivary cortisol was computed in CaPS and NSHD as the mean of waking and 30 min samples; to be comparable, in NCDS the cortisol measure taken within 45 minutes after waking was used. In HCS morning serum cortisol was used.

To be able to combine the cohorts in a meta-analysis, the cortisol values were standardized by deriving study-specific z-scores. In NCDS, both early and late morning cortisol were positively skewed, as was night time cortisol in CaPS and NSHD, so values were $\log_e$ transformed before they were converted to z-scores. In addition to the early morning and night time cortisol measures, we derived the diurnal slope (CaPS, NSHD, NCDS) as early morning value subtracted from the evening (or late morning in NCDS) value and divided by the lapsed time period. The overall slope is negative and so positive z-scores indicate a flatter response. The cortisol awakening response (CAR) was derived as the difference between 30 minute post waking sample and the waking sample (CaPS, NSHD).

Statistical Methods

A two stage meta-analysis was performed. In the first stage, we modelled WEMWBS as a function of each continuous diurnal cortisol indicator in turn using linear regression in each cohort separately, with adjustment for i) sex, age at cortisol measurement and follow-up time to the wellbeing measurement, ii) additional adjustment for BMI, smoking status and socioeconomic position. There was no evidence of deviation from linearity in the association

For peer review only - http://bmjopen.bmj.com/site/about/guidelines.xhtml

between any of the cortisol measures and WEMWBS. There was no evidence of interaction between sex and any of the cortisol measures. In sensitivity analysis to explore possible bias arising from missing covariates , we adjusted for sex and age at cortisol measurement and follow-up time i) using the maximum available sample with cortisol and wellbeing data (results not presented), ii) using the sample restricted to only those participants that had complete data on all covariates. Results did not materially differ for these two samples. In the second stage, cohort-specific estimates were pooled in random-effects meta-analyses[32] chosen a priori due to the expected heterogeneity between the different studies.

Sensitivity analyses

We corrected regression estimates for regression dilution bias arising from error in the measurement of cortisol. The reliability ratios were estimated by regressing the cortisol measure on day 2 on the measure on day 1 from CaPS data.[33] This yielded reliability ratios of 0.554, 0.349 and 0.430 for morning, evening and slope cortisol respectively. In addition, because both heightened and blunted CAR have been linked to raised disease risk, we tested whether wellbeing differed for those with a CAR in the top third or bottom third relative to the middle third.

RESULTS

The characteristics of the participants of the four cohorts in the analysis are shown in Table 1. Age at time of wellbeing measurement ranged from mean (sd) age of 50.7 (0.1) years in NCDS to 80.1 (3.9) years in CaPS. Mean wellbeing ranged from 49.5 (7.9) in NCDS to 53.3 (10.6) in CaPS and increased with mean age of the cohort. Mean early morning cortisol values were similar for the three cohorts (CaPS, NSHD, NCDS) that had measured salivary cortisol. Night

For peer review only - http://bmjopen.bmj.com/site/about/guidelines.xhtml

time cortisol values were also similar although diurnal slope was more negative (indicating greater decline) in NCDS than in NSHD and CaPS.

We found no evidence of an association between early morning cortisol and wellbeing in the individual cohorts in sex, age and follow-up time adjusted models. The overall pooled estimate was 0.02 (95% CI -0.17,0.21; p=0.8) and there was no evidence of heterogeneity in this association across studies (I^2=2.3; p for heterogeneity=0.4) (Table 2 and Figure 1). Further adjustment for all covariates did not affect the overall pooled estimate 0.01 (95% CI -0.22,0.24; I^2=18.0; p for heterogeneity=0.3).

Sex, age and follow-up time adjusted associations between evening cortisol and wellbeing in the individual cohorts were in the expected direction (i.e. higher evening cortisol was associated with lower wellbeing) in NSHD: -0.33 (95% CI -0.77,0.11); CaPS -0.98 (95% CI -2.03,0.07). This indicates a weak inverse association between evening cortisol and wellbeing (Table 2 and Figure 2). Adjustment for BMI, smoking and social class attenuated this association. Again, there was no evidence of heterogeneity across studies (I^2=19.1; p for heterogeneity =0.3).

In the pooled analysis a flatter diurnal slope was associated with poorer wellbeing though this was not statistically significant before or after adjustment for all covariates (Table 2 and Figure 3). Results excluding NCDS (based on decline in cortisol between early and late morning) were similar (0.02 (95% CI -0.41, 0.46)). In the sex, age and follow-up time adjusted and in the fully adjusted models, a higher CAR tended to be associated with lower wellbeing although the association did not approach statistical significance (Table 2 and Figure 4).

For peer review only - http://bmjopen.bmj.com/site/about/guidelines.xhtml

In sensitivity analysis, correcting for regression dilution bias, the sex, age and follow-up time adjusted associations between cortisol and wellbeing were 0.036, -1.347 and -0.163 for morning, evening and slope cortisol respectively. We found no evidence that wellbeing differed according to thirds of the CAR distribution.

DISCUSSION

Based on meta-analysis of individual participant data from four large cohort studies, we found that morning cortisol, diurnal slope and CAR were not associated with wellbeing but there was evidence that higher evening cortisol was prospectively associated with lower positive mental wellbeing in middle and older age. The magnitude of this association was small (-0.06 standard deviations in wellbeing) in crude analysis though up to -0.17 standard deviations in wellbeing accounting for possible regression dilution bias. This association was attenuated by the inclusion of BMI, smoking and socioeconomic position. It remains unclear whether obesity is secondary to HPA axis dysregulation so this may represent over-adjustment for a variable on the explanatory pathway. Perceived stress is also linked to lower socioeconomic position, smoking and cortisol levels [27, 30] and so isolating an association between cortisol and wellbeing independently of these factors needs to be interpreted with caution.

Evening cortisol is arguably the least affected by salivary sampling protocol deviations, which can bias associations between cortisol and wellbeing towards the null.[7] Furthermore, single-day sampling tends to bias cortisol estimates towards state rather than trait values,[31] which may also explain the lack of association with wellbeing up to eight years later. Only one of the included studies captured cortisol profiles on more than one day and this study (that is,

For peer review only - http://bmjopen.bmj.com/site/about/guidelines.xhtml

For peer review only

CaPS) only sampled cortisol on two days. We note that there was also a trend towards lower wellbeing among those with higher morning cortisol in CaPS though this did not attain statistical significance. Replication in additional studies with samples over multiple days is warranted. We examined inter-individual differences in cortisol in relation to wellbeing up to eight years after assessment of cortisol patterns. It is possible that an association between cortisol and wellbeing would only be evident over a shorter lag time. When measured on the same day, studies have found higher positive affect among those with lower cortisol output.[14,34,35] In addition, lower output in the first 45 minutes after waking[8] and total output across the day[10] has been associated with higher wellbeing over a period of 3-4 weeks. Where intensive study designs have been used to measure intra-individual change based on serial measurements of both cortisol and wellbeing repeated over multiple days, some studies find evidence of an inverse association between cortisol output and positive affect[11,13,36-38] though others do not.[9,15,16] Trait positive affect has been found to predict higher evening cortisol (in men only)[39] though we are not aware that a prospective association between baseline cortisol and subsequent mental wellbeing has been assessed. Future studies are warranted in order to examine the longitudinal association between mental wellbeing and cortisol sampled over multiple days to more accurately capture trait cortisol. In addition, cortisol samples taken at regular intervals throughout follow-up would enable us to identify how changes in HPA-axis activation, such as hypocortisolism as a response to chronic stress, might be related to mental wellbeing.

Though the evidence remains inconsistent at present, explanatory pathways include the over-activation of the HPA axis which interacts with the serotonin system and may ultimately result in serotonin depletion, increasing proneness to negative emotional states and reducing positive emotionality.[6,40,41] In addition, it has been proposed that the

For peer review only - http://bmjopen.bmj.com/site/about/guidelines.xhtml

energy-mobilising properties of cortisol may underlie an association with positive mental wellbeing and that this pathway may be less relevant for understanding an association between cortisol and mental ill health.[7] The evidence base is currently too limited to determine whether diurnal cortisol is related to different components of positive mental wellbeing and to negative mental health in the same way though there is some indication that wellbeing and illbeing do not show the same correlations with cortisol slope.[10] The current study used WEMWBS, designed to capture both hedonic and eudemonic wellbeing over the last two weeks. Future research might explore a wider set of hedonic and eudemonic components of mental wellbeing, as well as measures of mental ill health, in a single analytical sample to establish whether they show the same or different relationships with diurnal cortisol.

Other limitations should be acknowledged. There were differences in the protocol for the collection of cortisol. Morning serum cortisol was collected in one study and salivary cortisol in the remaining three. Among those studies with salivary cortisol, morning average cortisol was derived from samples taken at or before the typical peak (CaPS and NSHD) but after the typical peak in NCDS, and cortisol levels differ considerably during this period.[42] In addition, we excluded participants taking endocrine system medication in NCDS in contrast to participants on cortico-steroid medication in the other studies though the former approach is more conservative. Despite these differences we did not find evidence of between-study heterogeneity in the estimates from the meta-analysis (with all I^2 values below 21%). We used clock time but cortisol patterns are more closely anchored to waking time. Participants were instructed to provide samples at specified times post-waking but actual waking time was not recorded. In addition, a maximum of four samples per day were collected and additional measures may have provided more accurate assessment of diurnal cortisol, especially diurnal

For peer review only - http://bmjopen.bmj.com/site/about/guidelines.xhtml

slope. We did not consider factors that may modify the prospective association between cortisol and wellbeing, such as social support which has been shown to buffer the health impact of stress.[43]

Nevertheless, the current study also has strengths. It includes a large number of participants (ranging from 1756 to 7515 in each analysis and considerably larger than any previous study), in population-based samples, using harmonized measures of wellbeing and covariates. Individual participant data meta-analysis was used, which has advantages over aggregate meta-analysis including greater statistical power and standardization of the derivation of variables and analytical models.[44]

In summary, the findings from this meta-analysis do not provide support for the hypothesis that cortisol profiles indicative of disrupted HPA axis functioning has strong associations positive mental wellbeing in healthy middle-aged and older people. Of the four diurnal cortisol levels considered here, only evening cortisol showed a prospective association with wellbeing and only in minimally-adjusted analysis. However, cortisol was sampled on only one or two days and studies with samples across multiple days may find stronger associations if they better characterize cortisol patterns, though it seems likely that any associations, if found, will be of modest to moderate magnitude.

For peer review only - http://bmjopen.bmj.com/site/about/guidelines.xhtml

Contributor statement:
YBS, CC, CG, CP and DK conceptualised the study. MS, YBS, MG, MCG and RC identified the variables, cleaned the data and undertook data analysis. All authors contributed to reviewing and interpreting the results, commenting on the manuscript and approved the final version.

Competing interests:
Authors have no conflicts of interest to report.

Funding:
MS, RC and DK are supported by the UK Medical Research Council (Programme codes MRC_MC_UU_12019/1; MRC_MC_UU_12019/4; MRC_MC_UU_12019/5). The MRC National Survey of Health and Development is funded by the UK Medical Research Council.

HALCyon was funded by the New Dynamics of Ageing (RES-353-25-0001) and MG was supported by this grant.

Data collection of cortisol in NCDS at 45 years was funded by the United Kingdom Medical Research Council, grant G0000934. This research was supported by the National Institute for Health Research Biomedical Research Centre at Great Ormond Street Hospital for Children NHS Foundation Trust and University College London.

The funders had no role in the study design, data collection, data analysis, data interpretation, writing of the report or the decision to submit the article for publication.

Data sharing statement:
MRC National Survey of Health and Development data used in this publication are available to bona fide researchers upon request to the NSHD Data Sharing Committee via a standard application procedure. Further details can be found at http://www.nshd.mrc.ac.uk/data. doi: 10.5522/NSHD/Q101; doi:10.5522/NSHD/Q102.

All requests for collaboration on the Caerphilly Prospective Study are reviewed by an independent steering committee (http://www.bris.ac.uk/social-community-medicine/projects/caerphilly/collaboration/).

National Child Development Study data are available via registration with the UK Data Service.

The Hertfordshire Cohort Study has governance and access arrangements that comply with MRC data sharing policy. The survey data are accessible to bona fide researchers by contacting Professor Cyrus Cooper, Director of the MRC Lifecourse Epidemiology Unit at the University of Southampton, who can forward a collaborators' agreement.

Acknowledgements:
The authors thank Stephanie Black for her contribution to data collation, statistical analysis and interpretation of the results presented here.

For peer review only - http://bmjopen.bmj.com/site/about/guidelines.xhtml

The authors are grateful to the Centre for Longitudinal Studies (CLS), UCL Institute of Education for the use of these data and to the UK Data Service for making them available. However, neither CLS nor the UK Data Service bear any responsibility for the analysis or interpretation of these data.

For peer review only

For peer review only - http://bmjopen.bmj.com/site/about/guidelines.xhtml

REFERENCES

1. Herbert J, Goodyer IM, Grossman, AB, et al, Do corticosteroids damage the brain? *J Neuroendocrinol* 2006;18:393-411.

2. Kumari M, Badrick E, Sacker A, et al.2010a. Identifying patterns in cortisol secretion in an older population. Findings from the Whitehall II study. *Psychoneuroendocrinology* 2010;35:1091-9.

3. Lupien S, Lecours AR, Schwartz G, et al. Longitudinal study of basal cortisol levels in healthy elderly subjects: evidence for subgroups. *Neurobiol Aging* 1996;17:95–105.

4. Souza-Talarico, J. N. D., Marin, M. F., Sindi, S., & Lupien, S. J. (2011). Effects of stress hormones on the brain and cognition: evidence from normal to pathological aging. Dementia & Neuropsychologia, 5(1), 8-16.

5. Huppert FA, Whittington JE. Evidence for the independence of positive and negative well-being: implications for quality of life assessment. *Br J Health Psychol* 2003;8:107-22.

6. Braquehais MD, Picouto MD, Casas M, et al. Sher, L., 2012. Hypothalamic-pituitary-adrenal axis dysfunction as a neurobiological correlate of emotion dysregulation in adolescent suicide. World J. Pediatr. 8, 197-206.

7. Smyth N, Thorn L, Hucklebridge F, et al. Post awakening salivary cortisol secretion and trait well-being: The importance of sample timing accuracy. *Psychoneuroendocrinology* 2015;58:141-51.

8. Evans P, Forte D, Jacobs C, et al. Cortisol secretory activity in older people in relation to positive and negative well-being. *Psychoneuroendocrinology* 2007;32:922-930.

For peer review only - http://bmjopen.bmj.com/site/about/guidelines.xhtml

9. Adam EK, Hawkley LC, Kudielka BM, et al. Day-to-day dynamics of experience – cortisol associations in a population-based sample of older adults. *Proc Natl Acad Sci USA* 2006;103:17058-63.

10. Ryff CD, Love GD, Urry HL, et al. Psychological well-being and ill-being: do they have distinct or mirrored biological correlates? *Psychother Psychosom* 2006;75:85–95.

11. Smyth J, Ockenfels MC, Porter L, et al. Stressors and mood measured on a momentary basis are associated with salivary cortisol secretion. *Psychoneuroendocrinology* 1998;23:353–370.

12. Lai JC, Evans PD, Ng SH, et al. Optimism, positive affectivity, and salivary cortisol. *Br J Health Psychol* 2005;10:467-84.

13. Steptoe A, Wardle J. Positive affect and biological function in everyday life. *Neurobiol Aging* 2005;26 Suppl 1:108-12.

14. Steptoe A, Wardle J, Marmot M, et al. Positive affect and health-related neuroendocrine, cardiovascular, and inflammatory processes. *Proc Natl Acad Sci USA* 2005;102(18):6508-12.

15. van Eck M, Berkhof H, Nicolson N, et al. The effects of perceived stress, traits, mood states, and stressful daily events on salivary cortisol. *Psychosom Med* 1996;58:447-58.

16. Jacobs N, Myin-Germeys I, Derom C, et al. A momentary assessment study of the relationship between affective and adrenocortical stress responses in daily life. *Biol Psychol* 2007;74:60–66.

17. Porges SW. The polyvagal theory: New insights into adaptive reactions of the autonomic nervous system. *Cleve Clin J Med* 2009; 76(Suppl 2):S86–S90.

18. Kuh D, Cooper R, Richards M, et al. A life course approach to healthy ageing: the HALCyon programme. *Public Health* 2012;126:193-5.

For peer review only - http://bmjopen.bmj.com/site/about/guidelines.xhtml

19. The Caerphilly and Speedwell Collaborative Group. Caerphilly and Speedwell collaborative heart disease studies. *J Epidemiol Community Health* 1984;38:259–262.

20. Syddall HE, Aihie SA, Dennison EM, et al. Cohort profile: the Hertfordshire cohort study. *Int J Epidemiol* 2005;34:1234—1242.

21. Kuh D, Pierce M, Adams J, et al. Cohort Profile: updating the cohort profile for the MRC National Survey of Health and Development: a new clinic-based data collection for ageing research. *Int J Epidemiol* 2011;40:e1—e9.

22. Power C, Elliott J. Cohort profile: 1958 British birth cohort (National Child Development Study). *Int J Epidemiol* 2006;35:34-41.

23. Kirschbaum C, Hellhammer DH. Salivary cortisol in psychobiological research: an overview. *Neuropsychobiology* 1989;22:150—169.

24. Gale CR, Cooper R, Craig L, et al. Cognitive function in childhood and lifetime cognitive change in relation to mental wellbeing in four cohorts of older people. PLoS One 2012;7(9):e44860.

25. Tennant R, Hiller L, Fishwick R, et al. The Warwick-Edinburgh Mental Well-being Scale (WEMWBS): development and UK validation. *Health Qual. Life Outcomes* 2007;27(5):63.

26. Badrick E, Bobak M, Britton A, et al. The relationship between alcohol consumption and cortisol secretion in an aging cohort. *J Clin Endocrinol Metab* 2008;93:750–757.

27. Kumari M, Badrick E, Chandola T, et al. Measures of social position and cortisol secretion in an aging population: findings from the Whitehall II study. *Psychosom Med* 2010;72:27-34.

For peer review only - http://bmjopen.bmj.com/site/about/guidelines.xhtml

28. Geoffroy MC, Li L, Power C. Depressive symptoms and body mass index: co-morbidity and direction of association in a British birth cohort followed over 50 years. *Psychol Med* 2014;44:2641-2652.

29. Power C, Li L, Hertzman C. Cognitive development and cortisol patterns in mid-life: findings from a British birth cohort. *Psychoneuroendocrinology* 2008;33:530-9.

30. Reid JD, Intrieri RC, Susman EJ, et al. The relationship of serum and salivary cortisol in a sample of healthy elderly. *J Gerontol* 1992;47:176—179.

31. Adam EK, Kumari M. Assessing salivary cortisol in large-scale, epidemiological research. *Psychoneuroendocrinology* 2009;34:1423-36.

32. DerSimonian R, Laird N. Meta-analysis in clinical trials. *Control Clin Trials* 1986;7:177-88.

33. Berglund L. Regression dilution bias: Tools for correction methods and sample size calculation. *Upsala J Med Sci* 2012;117:279-83.

34. Steptoe A, Gibson EL, Hamer M, et al. Neuroendocrine and cardiovascular correlates of positive affect measured by ecological momentary assessment and by questionnaire. *Psychoneuroendocrinology* 2007;32:56-64.

35. Steptoe A, O'Donnell K, Badrick E, et al. Neuroendocrine and inflammatory factors associated with positive affect in healthy men and women: the Whitehall II study. *Am J Epidemiol* 2008;167:96-102.

36. Hoppmann CA, Klumb PL. Daily goal pursuits predict cortisol secretion and mood states in employed parents with preschool children. *Psychosom Med* 2006;68:887-94.

37. Davydov DM, Shapiro D, Goldstein IB, et al. Moods in everyday situations: effects of menstrual cycle, work, and stress hormones. *J Psychosom Res* 2005;58:343–349.

For peer review only - http://bmjopen.bmj.com/site/about/guidelines.xhtml

38. Simpson EE, McConville C, Rae, G., et al. Salivary cortisol, stress and mood in healthy older adults: the Zenith study. *Biol Psychol* 2008;78(1):1–9

39. Polk DE, Cohen S, Doyle WJ, et al. State and trait affect as predictors of salivary cortisol in healthy adults. *Psychoneuroendocrinology* 2005;30:261–272.

40. Dinan TG. Glucocorticoids and the genesis of depressive illness. A psychobiological model. *Br J Psych* 1994;164:365-71.

41. Stokes PE. The potential role of excessive cortisol induced by HPA hyperfunction in the pathogenesis of depression. *Eur Neuropsychopharm* 1995;77-82.

42. Vreeburg SA, Hoogendijk WJ, val Pelt J, DeRijk RH, Verhagen JC, van Dyck R et al. Major depressive disorder and hypothalamic-pituitary-adrenal axis activity: results from a large cohort study. *Arch Gen Psych* 2009;66:617-26.

43. Hostinar CE, Gunnar MR. Social Support Can Buffer against Stress and Shape Brain Activity. *AJOB Neurosci* 2015;6:34-42.

44. Riley RD, Lambert PC, Abo-Zaid G. Meta-analysis of individual participant data: rationale, conduct, and reporting. *Br Med J* 2010;340, 221.

For peer review only - http://bmjopen.bmj.com/site/about/guidelines.xhtml

Table 1: Descriptive statistics of participants by study

	CaPS	HCS	NSHD	NCDS
Sample with WEMWBS and cortisol; n	592	1055	1736	5337
Gender; n(%) male	530 (100)	463 (44)	809 (47)	2464 (50)
Age range at cortisol measurement (years); [mean (sd)]	65 – 82 [72.8 (4.0)]	60 – 73 [66.6 (2.7)]	62 – 64 [63.6 (0.8)]	44 – 46 [44.1 (0.2)]
Age range at WEMWBS assessment (years); [mean (sd)]	73 - 90 [80.1 (3.9)]	69 – 78 [73.2 (2.4)]	62 - 64 [63.6 (0.8)]	50 – 51 [50.7(0.1)]
Follow-up time cortisol to WEMWBS assessment (years); mean (sd)	7.6 (0.4)	6.6 (1.2)	0	6.6 (0.3)
BMI (kg/m^2); mean(sd)	28.2 (3.8)	26.9 (4.2)	27.9 (4.8)	27.1 (4.7)
Adult current smoking status; n (%) smoker	53 (11.0)	84 (8.1)	160 (10.2)	893 (18.5)
Adult social class; n(%) manual	303 (59.5)	400 (39)	499 (29.2)	1626 (34.1)
WEMWBS score; mean (sd)	53.3 (10.6)	51.7 (8.1)	51.8 (8.0)	49.5 (7.9)
Sampling times; mean				
Waking sample	7.37AM	N/A	7.11AM	N/A
Waking + 30mins (+45 mins for NCDS)	8.13AM		7.42AM	8.11AM
Waking + 3h 45 mins	N/A		N/A	11:11AM
2pm sample	2.11PM		N/A	N/A
Evening sample	10.05PM		9.27PM	N/A
Serum cortisol (nmol/L)	N/A	258.1 (81.2)	N/A	N/A
Salivary cortisol (nmol/L)				
Early morning; mean (sd)	19.7 (9.5)		22.9 (9.6)	21.3 (10.8)
Night time; median (IQR)	2.3 (1.5, 3.5)		2.4 (1.7,3.7)	N/A
Diurnal slope (nmol/L/h); mean (sd)	-1.13 (0.7)		-1.43 (0.7)	-4.3 (3.8)
Cortisol Awakening Response; mean (sd)	2.3 (9.4)		6.4 (11.7)	N/A
Exclusions due to being on corticosteroids; n(%)	62 (10%)	13 (1.2%)	19(1.1%)	396 (7.5)[a]

[a]endocrine medication

For peer review only - http://bmjopen.bmj.com/site/about/guidelines.xhtml

Table 2. Overall summary estimates of effect for the associations between cortisol measures and wellbeing from a series of meta-analyses.

	Included cohorts	Number of individuals	Mean difference in WEMWBS score (95% CI) per SD increase in cortisol							
			Model 1[a]				Model 2[b]			
			Regression coefficient (95% CI)	P-value	Tests of heterogeneity I^2(%)	P-value[c]	Regression coefficient (95% CI)	P-value	Tests of heterogeneity I^2(%)	P-value[c]
Early morning cortisol	All	7515	0.02 (-0.17,0.21)	0.8	2.3	0.4	0.01 (-0.22,0.24)	0.9	18.0	0.3
Evening cortisol	CaPS NSHD	1756	-0.47 (-1.00,0.05)	0.08	20.3	0.3	-0.31 (-0.83,0.21)	0.2	19.1	0.3
Flatter diurnal slope	CaPS NSHD NCDS	6490	-0.07 (-0.27,0.14)	0.5	0.0	0.6	-0.08 (-0.28,0.13)	0.5	0.0	0.6
Cortisol awakening response (CAR)	CaPS NSHD	1612	-0.19 (-0.62, 0.24)	0.4	0.0	1.0	-0.16 (-0.59,0.26)	0.4	0.0	0.9

[a]sex, age at cortisol assessment, follow-up time to wellbeing assessment; [b]Model 1 plus body mass index, smoking status, adult social class; [c]p-values from Cochran's Q statistic performed as a test of between-study heterogeneity

For peer review only - http://bmjopen.bmj.com/site/about/guidelines.xhtml

Figure Legend

Figure 1. Meta-analysis of the association between wellbeing and early morning cortisol (sex and follow-up time adjusted)

Figure 2. Meta-analysis of the association between wellbeing and evening cortisol (sex and follow-up time adjusted)

Figure 3. Meta-analysis of the association between wellbeing and flatter diurnal slope (sex and follow-up time adjusted)

Figure 4. Meta-analysis of the association between wellbeing and cortisol awakening (sex and follow-up time adjusted)

For peer review only

For peer review only - http://bmjopen.bmj.com/site/about/guidelines.xhtml

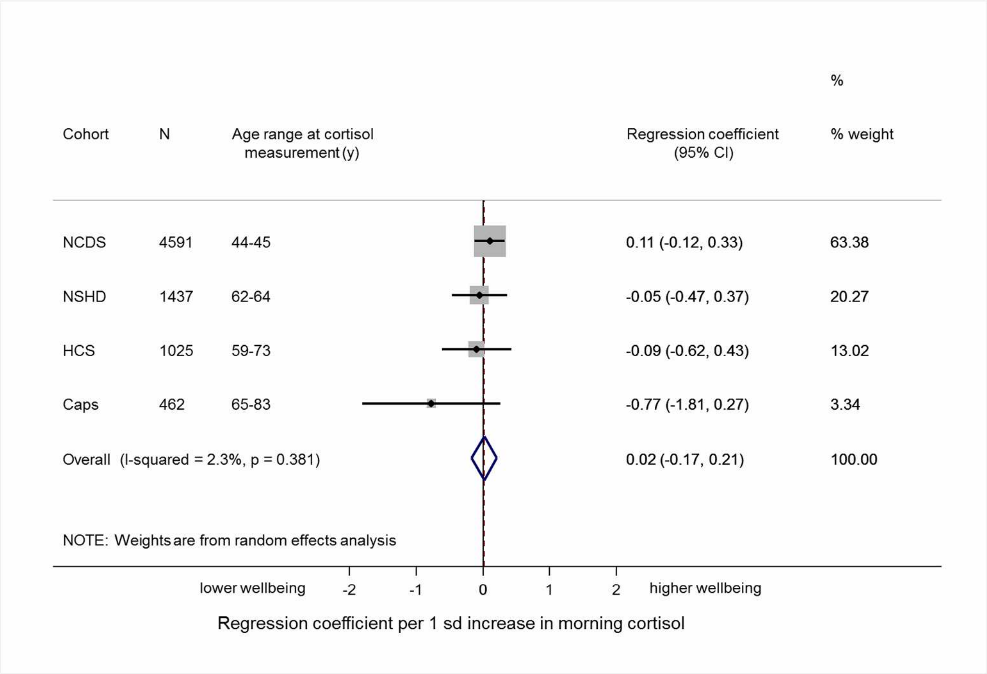

Figure 1

276x190mm (300 x 300 DPI)

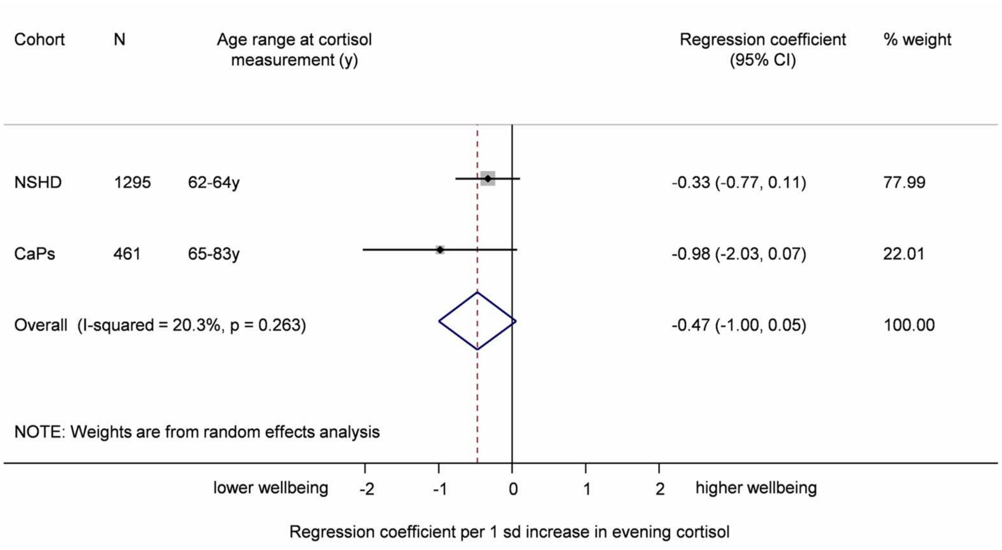

Figure 2

137x74mm (300 x 300 DPI)

review only

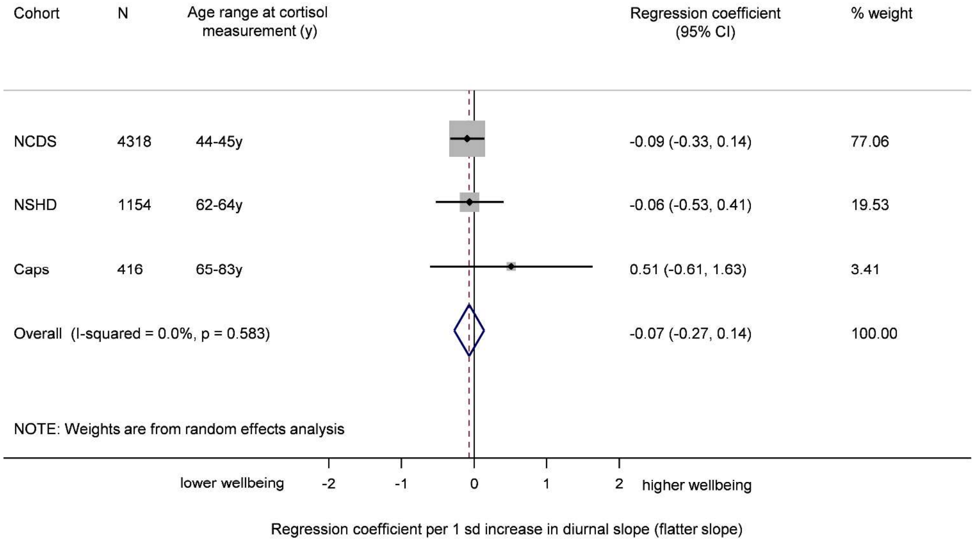

Figure 3

508x381mm (300 x 300 DPI)

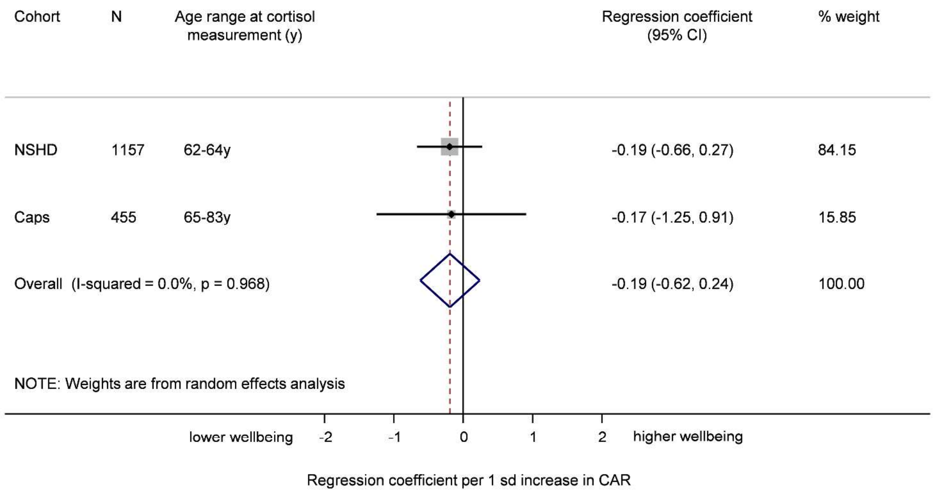

Figure 4

508x381mm (300 x 300 DPI)

STROBE Statement—checklist of items that should be included in reports of observational studies

	Recommendation	Page
**Title and abstract**	(*a*) Indicate the study's design with a commonly used term in the title or the abstract	1,2
	(*b*) Provide in the abstract an informative and balanced summary of what was done and what was found	1,2
**Introduction**		
Background/rationale	Explain the scientific background and rationale for the investigation being reported	4
Objectives	State specific objectives, including any prespecified hypotheses	5
**Methods**		
Study design	Present key elements of study design early in the paper	5
Setting	Describe the setting, locations, and relevant dates, including periods of recruitment, exposure, follow-up, and data collection	5,6
Participants	(*a*) *Cohort study*—Give the eligibility criteria, and the sources and methods of selection of participants. Describe methods of follow-up	5,6
	(*b*) *Cohort study*—For matched studies, give matching criteria and number of exposed and unexposed	N/A
Variables	Clearly define all outcomes, exposures, predictors, potential confounders, and effect modifiers. Give diagnostic criteria, if applicable	6-8
Data sources/ measurement	For each variable of interest, give sources of data and details of methods of assessment (measurement). Describe comparability of assessment methods if there is more than one group	5-7
Bias	Describe any efforts to address potential sources of bias	9-10
Study size	Explain how the study size was arrived at	9, Table 1
Quantitative variables	Explain how quantitative variables were handled in the analyses. If applicable, describe which groupings were chosen and why	10
Statistical methods	(*a*) Describe all statistical methods, including those used to control for confounding	10-11
	(*b*) Describe any methods used to examine subgroups and interactions	11
	(*c*) Explain how missing data were addressed	11
	(*d*) *Cohort study*—If applicable, explain how loss to follow-up was addressed	
	(*e*) Describe any sensitivity analyses	11

Continued on next page

For peer review only - http://bmjopen.bmj.com/site/about/guidelines.xhtml

Results		Page
Participants	(a) Report numbers of individuals at each stage of study—eg numbers potentially eligible, examined for eligibility, confirmed eligible, included in the study, completing follow-up, and analysed	9, Table 1
	(b) Give reasons for non-participation at each stage	
	(c) Consider use of a flow diagram	N/A
Descriptive data	(a) Give characteristics of study participants (eg demographic, clinical, social) and information on exposures and potential confounders	Table 1
	(b) Indicate number of participants with missing data for each variable of interest	
	(c) *Cohort study*—Summarise follow-up time (eg, average and total amount)	
Outcome data	*Cohort study*—Report numbers of outcome events or summary measures over time	Table 1
Main results	(*a*) Give unadjusted estimates and, if applicable, confounder-adjusted estimates and their precision (eg, 95% confidence interval). Make clear which confounders were adjusted for and why they were included	Table 2
	(*b*) Report category boundaries when continuous variables were categorized	
	(*c*) If relevant, consider translating estimates of relative risk into absolute risk for a meaningful time period	
Other analyses	Report other analyses done—eg analyses of subgroups and interactions, and sensitivity analyses	12
**Discussion**		
Key results	Summarise key results with reference to study objectives	13
Limitations	Discuss limitations of the study, taking into account sources of potential bias or imprecision. Discuss both direction and magnitude of any potential bias	14
Interpretation	Give a cautious overall interpretation of results considering objectives, limitations, multiplicity of analyses, results from similar studies, and other relevant evidence	16
Generalisability	Discuss the generalisability (external validity) of the study results	13-14
**Other information**		
Funding	Give the source of funding and the role of the funders for the present study and, if applicable, for the original study on which the present article is based	17

.

For peer review only
